# Catecholamine-mediated release of miR-133a-3p from adipocytes regulates the onset of chronic primary pain

**DOI:** 10.1172/JCI197345

**Published:** 2026-07-21

**Authors:** Nathaniel P. Hernandez, Jiegen Chen, Yiling Qian, Xin Zhang, Yaomin Wang, Brittney P. Ciszek, Xianglong Gao, Marguerita E. Klein, Yun-Ling Pai, Mohamad Karaky, Carolina Beraldo Meloto, Francesca Montagna, Matt Kanke, Clair Crewe, Luda Diatchenko, Praveen Sethupathy, Andrea G. Nackley

**Affiliations:** 1 Center for Translational Pain Medicine, Department of Anesthesiology, and; 2 Department of Pharmacology and Cancer Biology, Duke University School of Medicine, Durham, North Carolina, USA.; 3 WhiteCap Implant Institute, Herber City, Utah, USA.; 4 Department of Cell Biology & Physiology, Washington University School of Medicine in St. Louis, St. Louis, Missouri, USA.; 5 Faculty of Medicine and Health Sciences, The Alan Edwards Centre for Research on Pain, and; 6 Faculty of Dental Medicine and Oral Health Sciences, Department of Anesthesia, McGill University, Montreal, Quebec, Canada.; 7 Department of Biomedical Sciences, Cornell University College of Veterinary Medicine, Ithaca, New York, USA.

**Keywords:** Endocrinology, Neuroscience, Adipose tissue, Gene therapy, Pain

## Abstract

Chronic primary pain conditions (CPPCs), such as fibromyalgia and vestibulodynia, affect over 100 million Americans, predominantly women, and pose a substantial healthcare challenge. CPPCs arise from genetic and environmental factors that enhance catecholamine tone, potentially through miRNA dysregulation following catecholamine activation of β-adrenergic receptors. Here, we identified miR-133a-3p as a biomarker of CPPC status and investigated its functions using in vivo and in vitro approaches. Plasma levels of miR-133a-3p were consistently downregulated in humans with ≥ 1 CPPC and in rat and mouse models of primary pain. Our data suggest that miR-133a-3p is packaged in extracellular vesicles that are secreted by adipocytes and trafficked to the spinal cord. Activation of adrenergic receptors on white adipocytes resulted in downregulation of miR-133a-3p, negatively regulating pain-related genes in the spinal cord, such as *MAP3K3*, which is critical for sensory neuron activation. Adipose-specific overexpression of miR-133a-3p in a mouse model of primary pain reversed mechanical hypersensitivity in both sexes. These findings implicate miR-133a-3p dysregulation in primary pain across conditions and species and establish its role in multisite mechanical hypersensitivity. Furthermore, miR-133a-3p overexpression shows therapeutic potential for the millions of individuals with CPPCs.

## Abstract 2

We discovered a small molecule released from fat cells, miR-133a-3p, that may help reduce chronic pain by calming nerve activity in the spinal cord.

## Graphical abstract

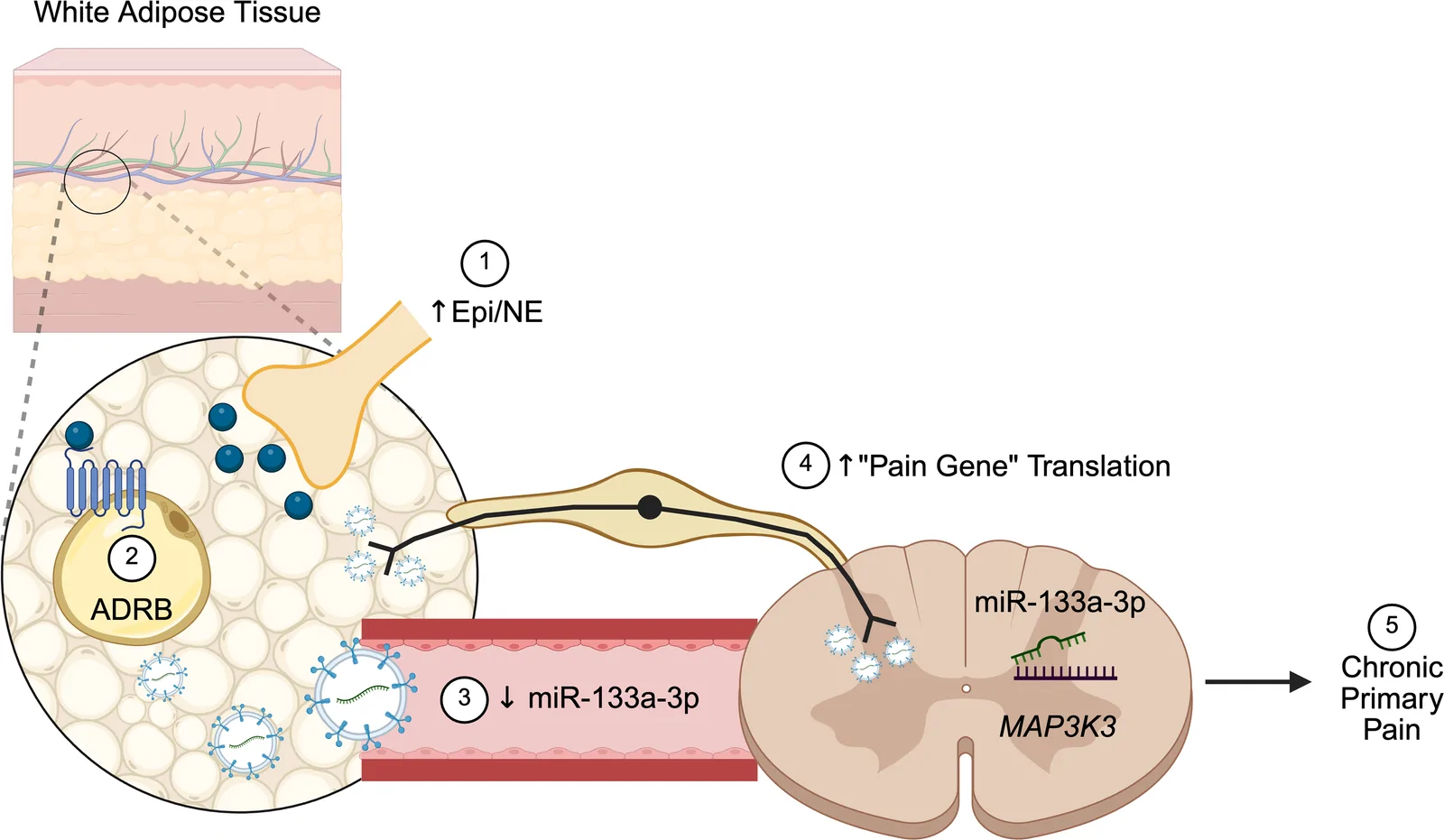

## Introduction

Nearly 1 in 3 Americans is affected by chronic primary pain conditions (CPPCs). Fibromyalgia syndrome (FMS), vestibulodynia (VBD), and chronic low-back pain are among the most common CPPCs. These conditions have also been termed chronic overlapping pain conditions because they frequently co-occur, and affected individuals often experience pain across multiple body sites (1). Current treatments, including opioids and anticonvulsants, have harmful side effects and fail to address disease etiology (2). Chronic pain has been the leading cause of adult disability for decades, yet few innovative therapies have emerged. Improved pain management for CPPCs may lie in leveraging their shared genetic and environmental stress signatures.

CPPCs are linked to loss-of-function mutations in the *COMT* gene, which encodes catechol-*o*-methyltransferase (COMT), an enzyme that metabolizes catecholamines (3, 4). Individuals with the low COMT activity genotype report greater pain at baseline and after stressful events that potentiate catecholamine release from sympathetic nerves (5–8). Consistent with clinical syndromes, our lab showed that delivery of the COMT inhibitor OR486 together with repeated swim stress produces pain at multiple body sites in rodents (9–11). This hypersensitivity is driven by activation of peripheral adrenergic receptor beta-2 (ADRB2) and ADRB3 (12) and their downstream effectors, including proinflammatory cytokines (10, 13) and, as shown here, miRNAs.

miRNAs are small noncoding RNAs that inhibit target mRNA translation. During stress, miRNAs are secreted from cells into extracellular vesicles (EVs) (14), which are transported to distant sites where miRNAs inhibit multiple mRNAs with complementary sequences (15–17). miRNA dysregulation contributes to chronic pain following nerve injury (15), inflammation (18), and trauma (19, 20), but its role in CPPCs remains poorly understood. We previously identified differential expression of circulating miRNAs in women with VBD and comorbid CPPCs affecting remote body sites (21). Yet, tissue-specific dysregulation requires investigation since tissues such as white adipose tissue (WAT) differentially contribute to circulating miRNAs (22, 23), and basal gene expression differs across cell types (24, 25). Consequently, a single miRNA may regulate distinct pathways depending on target availability within a tissue. We therefore sought to identify catecholamine-regulated miRNAs and test the hypothesis that they directly influence mechanical hypersensitivity at multiple body sites.

This study identified miR-133a-3p as a catecholamine-regulated miRNA that is downregulated following catecholamine stimulation. This signature was validated in 2 clinical CPPC cohorts (VBD and FMS) and correlated with pain intensity in a dose-dependent manner in the FMS cohort. Using a rodent model of primary pain, we demonstrated that (a) WAT is a key source of miR-133a-3p downregulation; (b) increasing miR-133a-3p expression inhibits sensory neuron activation, likely through inhibition of pain-relevant targets, including *MAP3K3*; and (c) although miR-133a-3p is enriched in adipocyte-derived EVs, catecholamine stimulation reduced its EV abundance. Consistent with this, conditioned media from norepinephrine-treated adipocytes sensitized neuronal responsiveness to capsaicin. Importantly, adipocyte-targeted restoration of miR-133a-3p expression reversed mechanical hypersensitivity at multiple body sites in both male and female mice. Together, these findings suggest that loss of miR-133a-3p in adipocyte-derived EVs promotes sensory neuron sensitization through adipocyte-neuron crosstalk and reveal a direct role for adipocytes and miR-133a-3p in CPPC etiology.

## Results

### Sustained increases in catecholamine tone led to miRNA dysregulation.

**Figure 1:**
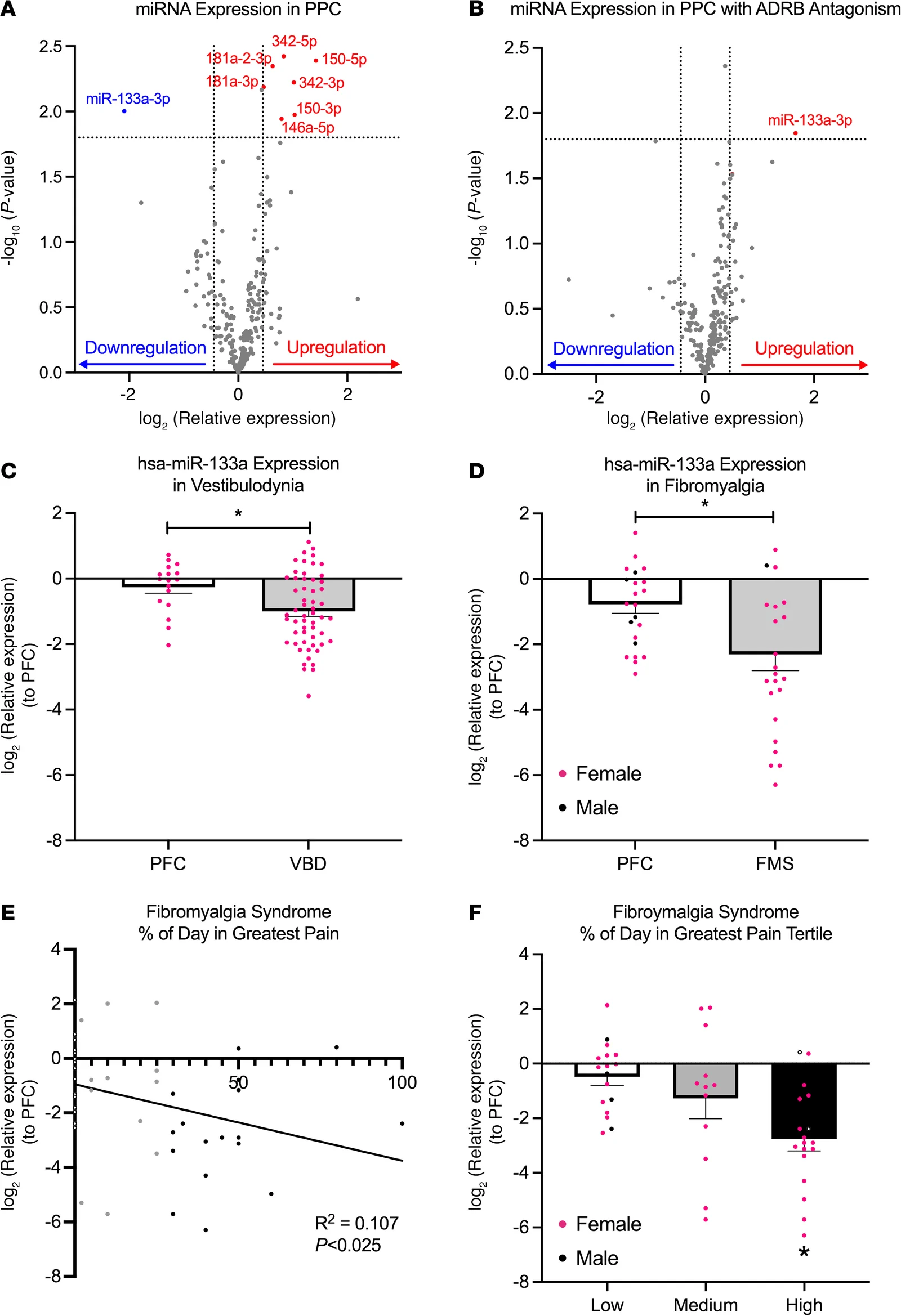
ADRB-dependent miR-133a-3p downregulation in plasma was a signature of CPPCs. (**A**) Compared with PFCs, rats in the PPC model exhibited significant upregulation of 7 miRNAs and downregulation of 1 miRNA (miR-133a-3p). (**B**) Compared with the PPC group receiving vehicle, those receiving the ADRB2 and ADRB3 antagonists ICI-118,511 and SR59230A exhibited significant upregulation of miR-133a-3p. *N* = 5/group. Differential expression determined by small RNA-seq with Benjamini-Hochberg–adjusted *P* < 0.05. (**C**) Plasma miR-133a-3p levels were reduced in women with VBD compared with PFCs. *N* = 67 (22 PFC, 45 VBD). (**D**) Plasma miR-133a-3p levels were reduced in individuals with FMS compared with PFCs. *N* = 48 (24 PFC, 24 FMS). (**E**) Plasma miR-133a-3p levels were inversely associated with the percentage of the day spent at peak pain severity in the FMS cohort. Data points colored by tertile. (**F**) Individuals in the highest tertile of time spent at peak pain severity exhibit lower plasma miR-133a-3p levels than those in the lowest tertile. Data are presented as the mean + SEM. Data in **C**, **D**, and **F** were analyzed by ANOVA with Tukey’s HSD post hoc test. *P < 0.05.

We first sought to identify miRNAs dysregulated in a rodent model of primary pain conditions (PPCs) in an ADRB2/3-dependent manner. PPC female rats were implanted with subcutaneous osmotic minipumps delivering the COMT inhibitor OR486 and a second minipump delivering the ADRB2- and ADRB3-selective antagonists ICI-118,511 and SR59230A or corresponding vehicle for 14 days. Pain-free controls (PFCs) received 2 minipumps delivering the corresponding vehicles. On day 14, whole blood was collected for small RNA isolation and library preparation. Small RNA-seq revealed that the most prevalent small RNA length was 22 nucleotides (26.63% of reads), the typical size for miRNAs (14). Compared with PFC rats, PPC rats had significantly higher levels of 7 miRNAs (miR-146a-5p, miR150-5p, miR-150-3p, miR-181a-3p, miR-181a-2-3p, miR-342-5p, and miR-342-3p) and significantly lower levels of 1 miRNA (miR-133a-3p) (*P* < 0.05; Figure 1A). Compared with PPC rats, PPC rats receiving ADRB2/3 antagonists had significantly higher miR-133a-3p levels (*P* < 0.05; Figure 1B), suggesting that reduced miR-133a-3p is dependent on catecholamine activation of ADRB2 and ADRB3. Unlike other significant miRNAs, miR-133a-3p may play a mechanistic role in the CPPC development.

### Validation of miR-133a-3p reduction in plasma from individuals with CPPCs.

Next, we validated miR-133a-3p downregulation in 2 CPPC cohorts with a primary diagnosis of VBS or FMS. Compared with PFCs, plasma from women with VBD (*P* < 0.002; Figure 1C) and individuals with FMS (*P* < 0.011; Figure 1D) exhibited significantly lower miR-133a-3p levels. Pain data were collected in the FMS cohort. Linear regression demonstrated that lower miR-133a-3p levels were significantly associated with a greater percentage of the day spent at peak pain severity. Approximately 11% of the variability in peak pain severity was explained by miR-133a-3p levels (*R*^2^ = 0.107, *P* < 0.025; Figure 1E). When participants were stratified into tertiles based on time spent at peak pain severity, those in the highest tertile exhibited significantly lower plasma miR-133a-3p levels than those in the lowest tertile (*P* < 0.003; Figure 1F).

### Tissue signature of miR-133a-3p levels in mice after an acute or sustained increase in catecholaminergic tone.

**Figure 2:**
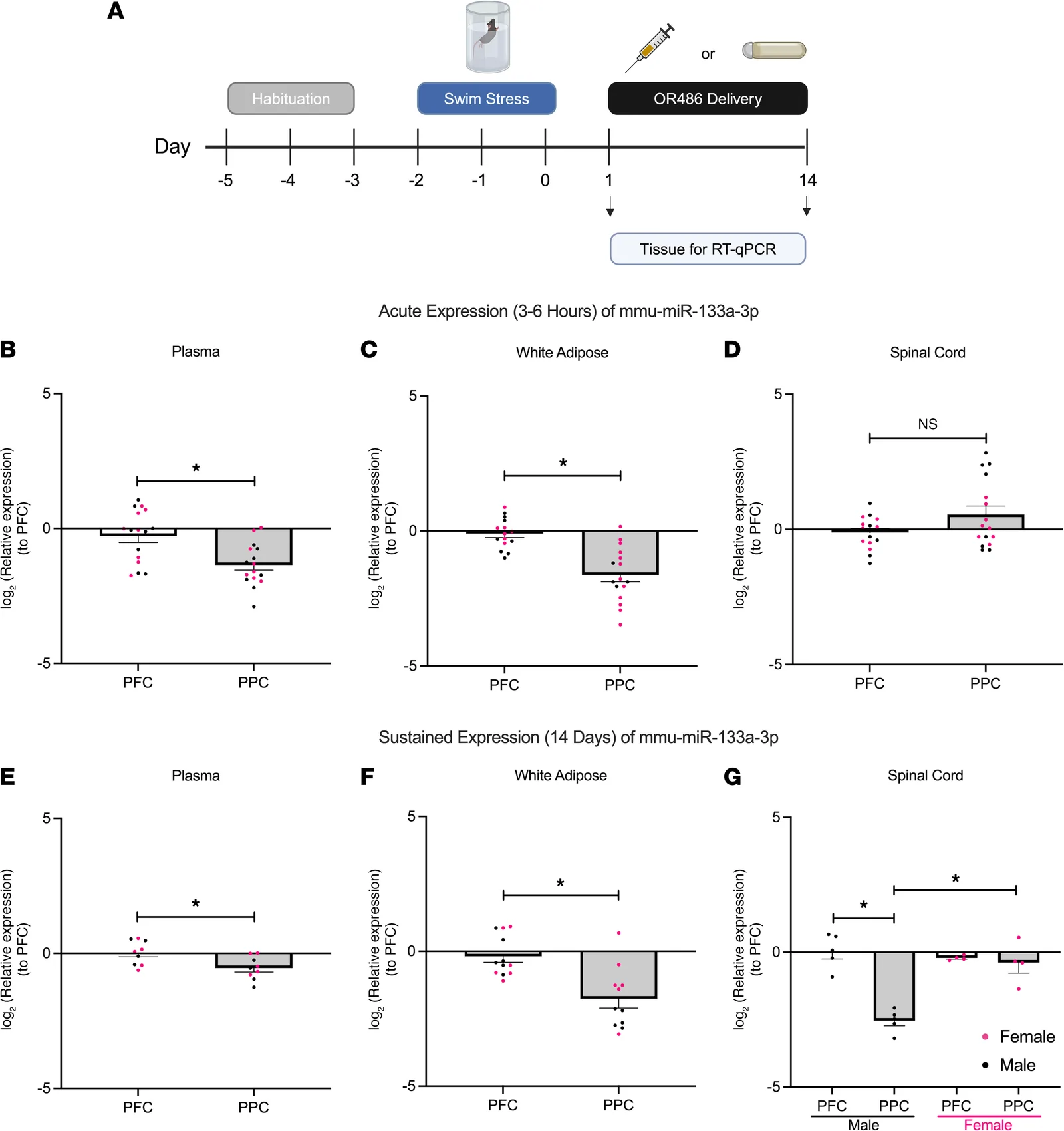
miR-133a-3p downregulation likely originated in WAT. (**A**) Schematic of the treatment timeline. (**B**–**G**) Compared with PFCs, PPC mice had significantly lower levels of miR-133a-3p at acute (3–6 hours) and sustained (14 days) time points in plasma (**B** and **E**) and WAT (**C** and **F**). (**D** and **G**) In the spinal cord, levels were unchanged at the acute time point but were lower in males only at the sustained time point. *N* = 4–8 mice/sex/group. Data are presented as the mean + SEM. Data in **B**–**G** were analyzed by ANOVA followed by Tukey’s HSD post hoc test. *P < 0.05.

To identify the source of miR-133a-3p downregulation, we screened 7 tissues (plasma, white adipose, brown adipose, skeletal muscle, liver, spinal cord, and dorsal root ganglion [DRG]) from male and female mice following acute (3–6 hours) or sustained (14 days) OR486 delivery (Figure 2A). We shifted from rats to mice because mouse models are well suited for genetic manipulation (26). We evaluated the acute time point because miRNA signatures measured shortly after traumatic stress, such as <24 hours following a motor vehicle collision or <72 hours following sexual assault trauma, can predict transition to chronic pain (20). miR-133a-3p is expressed in adipose (27), nervous (28, 29), and skeletal muscle tissue (30), where it has been linked to pain in other mouse models (31–33). Notably, the mature 22-nucleotide miR-133a-3p sequence is completely conserved among humans, rats, and mice (34) (Supplemental Figure 1; supplemental material available online with this article; https://doi.org/10.1172/JCI197345DS1).

Compared with PFCs at the acute time point, miR-133a-3p levels were significantly lower in PPC mice plasma (*P* < 0.003; Figure 2B) and white adipose (*P* < 0.013; Figure 2C), but not spinal cord (*P* < 0.478; Figure 2D), brown adipose (*P* < 0.282), muscle (*P* < 0.205), or DRG (*P* < 0.055) (Supplemental Figure 2). At the sustained time point, miR-133a-3p levels remained significantly lower in PPC mice plasma (*P* < 0.012; Figure 2E) and white adipose for both sexes (*P* < 0.001; Figure 2F) and in spinal cord in males only (treatment × sex, *P* < 0.001, Tukey’s HSD post hoc, *P* < 0.001; Figure 2G). No differences were observed in brown adipose (*P* < 0.056), muscle (*P* < 0.139), DRG (*P* < 0.919), or liver (*P* < 0.975) at the sustained time point (Supplemental Figure 2). These findings validate the rat and human cohorts and identify WAT as a likely source of miR-133a-3p downregulation that may contribute to reduced circulating levels and, in males, reduced spinal cord levels by 14 days.

### Tissue signature of pre-miR-133a-3p expression in mice after an increase in catecholaminergic tone.

To further evaluate white adipocytes as a source of miR-133a-3p downregulation, we quantified pre-miR-133a-3p expression. Since extracellular precursor miRNAs are rarely detected (35), their presence is more likely to reflect endogenous miRNA synthesis. Compared with PFCs, pre-miR-133a-3p levels in WAT trended toward downregulation in PPC mice (*P* < 0.065), but not in spinal cord or liver (*P* < 0.776 and *P* < 0.581, respectively), and were not detected in the plasma of either group (Supplemental Figure 3).

### Catecholamine stimulation of white adipocytes drove miR-133a-3p downregulation intracellularly and in secreted EVs.

**Figure 3:**
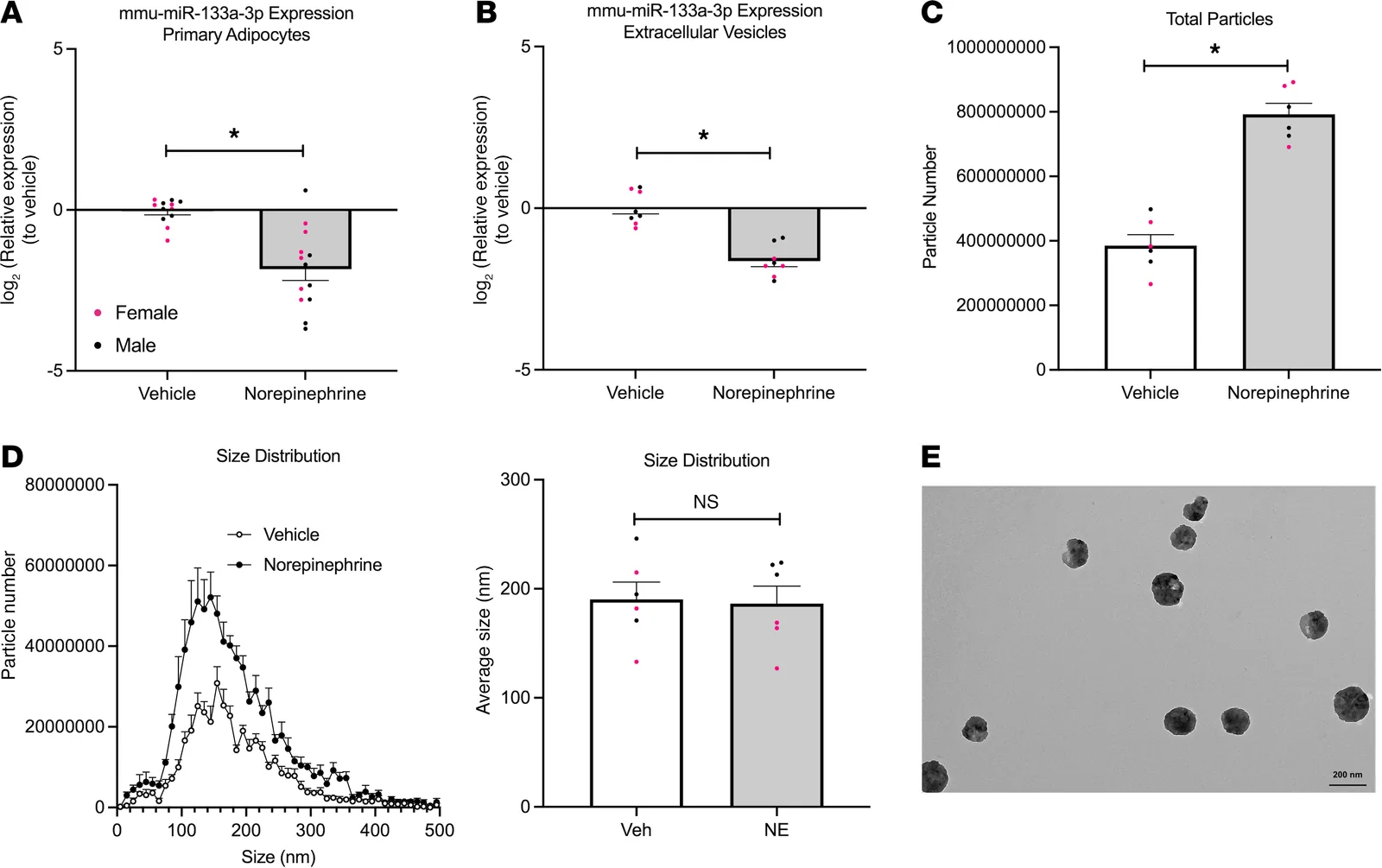
Adrenergic receptor stimulation caused miR-133a-3p downregulation in both primary white adipocytes and their secreted EVs. (**A** and **B**) Compared with vehicle treatment, norepinephrine treatment for 3 hours reduced miR-133a-3p levels in primary white adipocytes (**A**) and EVs (**B**) secreted into culture media. (**C** and **D**) Norepinephrine-treated adipocytes released significantly more EVs, (**C**) and EV size did not differ between groups (**D**). (**E**) Representative electron microscopy image of secreted EVs. Scale bar: 200 nm. *N* = 3–7 wells/sex/group. Data are presented as the mean + SEM. Data in **A**–**D** analyzed by ANOVA followed by Tukey’s HSD post hoc test. *P < 0.05.

To directly test the effects of catecholamines on miR-133a-3p expression, we cultured primary white adipocytes and DRG neurons from male and female mice and treated them with norepinephrine. Preadipocytes were differentiated into mature white adipocytes before experimentation. Compared with vehicle-treated controls, norepinephrine-treated adipocytes showed significant downregulation of miR-133a-3p (*P* < 0.001; Figure 3A). In contrast, miR-133a-3p levels were unchanged in neuronal cultures (Supplemental Figure 4). Next, we isolated EVs from adipocyte culture media and found significantly lower levels of miR-133a-3p in EVs secreted from norepinephrine-treated adipocytes (*P* < 0.001; Figure 3B). Norepinephrine increased total EV secretion (*P* < 0.001; Figure 3C), but EV size was unchanged between groups and remained within the expected range for adipocyte EVs (average, 188 nm; *P* < 0.869; Figure 3, D and E). These data support adipocytes as the source of miR-133a-3p downregulation and suggest that miR-133a-3p–deficient EVs may alter regulation of pain-relevant genes in distant tissues.

### Adipose-targeted overexpression of miR-133a-3p reversed mechanical hypersensitivity in a mouse model of PPC.

**Figure 4:**
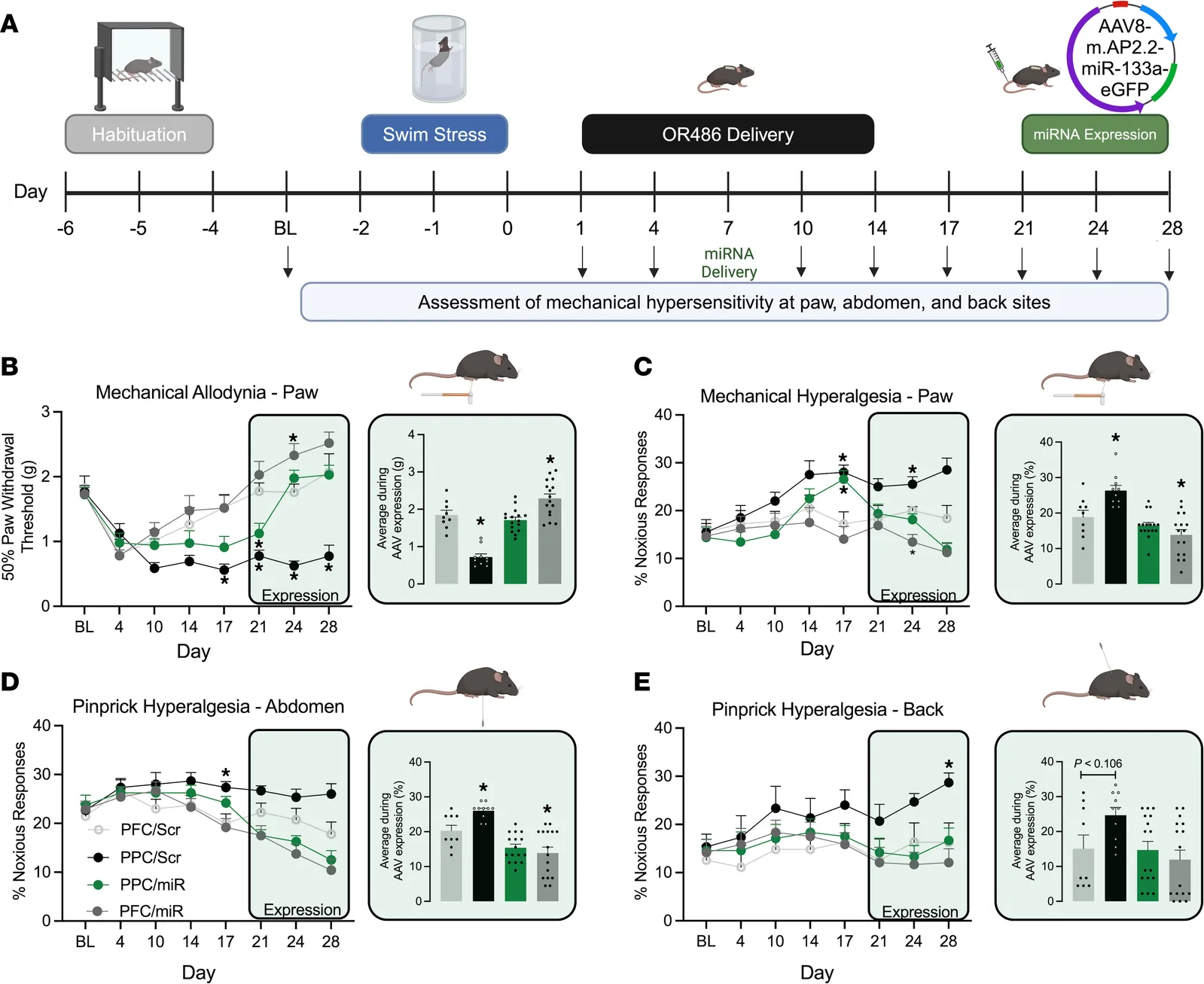
Adipose-specific overexpression of miR-133a-3p reversed multisite mechanical sensitivity in a rodent model of primary pain. (**A**) Timeline for behavioral assays and delivery of rAAV-m.AP2.2-miR-133a-3p-eGFP or scramble control vector. (**B**–**E**) Compared with PFC/scramble mice, PFC/miR-133a-3p mice exhibited increased tolerance to mechanical stimuli in the paw and abdomen. Compared with PPC/scramble mice, PPC/miR-133a-3p mice exhibited reversal of mechanical hypersensitivity during active vector expression (days 21–28). During this period, PPC/miR-133a-3p mice were statistically indistinguishable from PFC/scramble controls. *N* = 3–8 mice/sex/group. Data are presented as the mean + SEM. Data in **B**–**E** were analyzed by mixed-effects model with repeated measures and Dunnett’s multiple-comparison test. Bar graph callouts were analyzed by 3-way ANOVA followed by Dunnett’s multiple-comparison test. *P < 0.05 versus PFC/scramble at matched time points.

Next, we tested whether adipocyte-targeted overexpression of miR-133a-3p could reverse primary pain. We delivered rAAV8-mAP2.2-miR-133a-3p-eGFP or scramble vector via tail vein injection 7 days following PPC model induction. Mechanical allodynia and hyperalgesia were assessed at multiple body sites (paw, abdomen, and back) at baseline and over 28 days. A timeline is shown in Figure 4A.

For all behavioral assays, mixed-effects models with repeated measures and appropriate post hoc testing were used to assess group, treatment, and sex effects on outcome variables. In the 50% paw withdrawal threshold assay (mechanical allodynia), PPC/scramble mice exhibited significantly lower thresholds than PFC/scramble mice at matched time points. As expected, male and female PPC/miR-133a-3p mice also exhibited reduced thresholds at early time points (days 4–17). However, after active vector expression was achieved approximately 2 weeks after injection (days 21–28), thresholds in PPC/miR-133a-3p mice were indistinguishable from PFC/scramble mice. Moreover, PFC/miR-133a-3p mice exhibited significantly increased thresholds compared with PFC/scramble mice during active vector expression (*P* < 0.05 at matched time points; Figure 4B). Similar patterns were observed for paw mechanical hyperalgesia (Figure 4C), abdominal pinprick hyperalgesia (Figure 4D), and back pinprick hyperalgesia (all *P* < 0.05 vs. PFC/scramble at matched time points; Figure 4E). No main effects of sex were observed, although sex-stratified data are presented in Supplemental Figure 5, A–H. Together, these findings support adipocyte-specific miR-133a-3p as an analgesic capable of reversing primary pain in both sexes.

### Adipose-targeted overexpression of miR-133a-3p did not impair motor function.

To distinguish analgesic effects from altered motor performance, we used the Rotarod apparatus to compare latency to fall between mice treated with scramble or miR-133a-3p adeno-associated virus (AAV) at baseline and 28 days after injection. No differences were observed between treatment groups or sexes (*P* < 0.241; Supplemental Figure 6).

### Validation of rAAV8-mAP2.2-miR-133a-3p-eGFP delivery.

To validate efficient and specific transduction of the recombinant viral vector, we performed a presence/absence test for eGFP by RT-qPCR in adipose, spinal cord, and muscle collected on day 28 after behavioral testing. eGFP exceeded the detection threshold (Ct ≤ 36) in 88% of WAT samples but in only 8% and 4% of spinal cord and muscle samples, respectively (Supplemental Table 1). Mice receiving the miR-133a-3p vector also exhibited significantly increased miR-133a-3p levels in plasma, WAT, and spinal cord (all *P* < 0.05; Supplemental Figure 7, A–C), but not muscle (*P* < 0.102; Supplemental Figure 7D). To determine the time of viral expression, we used IHC to detect eGFP in a separate cohort collected at 2, 3, and 4 weeks after tail vein injection. Mice receiving the viral vector exhibited eGFP^+^ cells in all 3 adipose sources (inguinal white, perigonadal white, and brown adipose) at all time points examined. In contrast, eGFP^+^ cells were not observed in adipose tissue from PBS-treated mice or in muscle from AAV-treated mice 2 weeks after delivery (Supplemental Figure 7, E–G). Together, these findings support efficient and adipocyte-specific transduction of the recombinant viral vector.

### miR-133a-3p regulation of pain-relevant mRNA targets in neurons.

**Figure 5:**
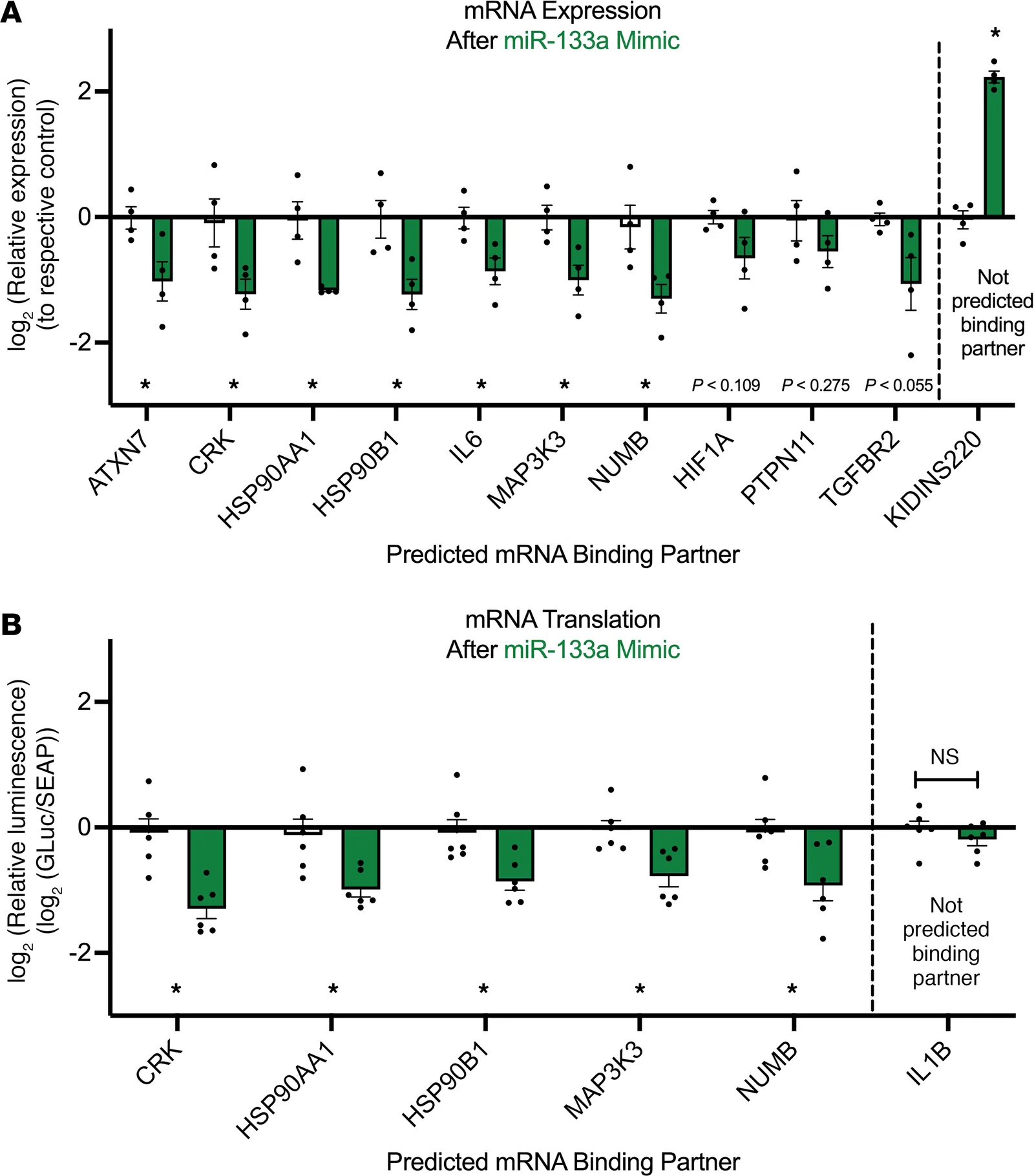
miR-133a-3p regulated the expression of pain-relevant mRNA targets in vitro. (**A**) Compared with scramble treatment, miR-133a-3p treatment significantly reduced expression of several predicted mRNA targets. *KIDINS220*, which is not a predicted target, was significantly upregulated. (**B**) miR-133a-3p treatment inhibited translation of several predicted mRNA targets, but not *IL1B*, which is not a predicted target. *N* = 4–6 biological replicates/gene. Data are presented as the mean + SEM. Data were analyzed by ANOVA followed by Tukey’s HSD post hoc test. *P < 0.05.

To identify downstream mRNA targets through which miR-133a-3p may regulate nociception, we screened predicted miRNA-mRNA binding partners using established databases. From the hundreds of potential mRNA binding partners identified by TargetScan, miRwalk (36), and DIANA-TarBase (37), candidates were prioritized based on appearances across databases, prediction strength, and relevance to pain biology. The top 10 predictions were screened by RT-qPCR to assess the effects of miR-133a-3p on neuronal mRNA expression. Compared with scramble-transfected cultures, neurons treated with a miR-133a-3p mimic exhibited significant downregulation of 7 genes: *ATXN7* (*P* < 0.031), *CRK* (*P* < 0.046), *HSP90AA1* (*P* < 0.010), *HSP90B1* (*P* < 0.021), *IL6* (*P* < 0.021), *MAP3K3* (*P* < 0.018), and *NUMB* (*P* < 0.035). Three predicted targets, *HIF1A* (*P* < 0.109), *PTPN11* (*P* < 0.275), and *TGFBR2* (*P* < 0.055), were not significantly altered but trended toward downregulation. As a negative control, we measured *KIDINS220*, a gene associated with primary pain (38) but not predicted to bind to miR-133a-3p. Rather than being downregulated, *KIDINS220* was significantly upregulated following miR-133a-3p treatment (*P* < 0.001; Figure 5A), supporting target specificity.

The 5 most strongly downregulated targets (*CRK*, *HSP90AA1*, *HSP90B1*, *MAP3K3*, and *NUMB*) were advanced to a dual-luciferase assay to assess the effects of miR-133a-3p on target translation (39). *IL1B*, which is not predicted to bind to miR-133a-3p, was included as a negative control. Since miRNAs commonly bind the 3′UTR of target mRNAs (17), luciferase reporters containing the target 3′UTR were cotransfected into HEK293 cells with either miR-133a-3p mimic or scramble control. STarMir prediction software (40) predicted miR-133a-3p binding to all 5 targets, with logistic probabilities ranging from 0.576 (*CRK*) to 0.829 (*HSP90AA1*) (Supplemental Table 2). The same NCBI RefSeq-derived 3′UTR sequence was used for both STarMir predictions and luciferase constructs. Compared with scramble treatment, miR-133a-3p significantly reduced translation of all 5 targets (all *P* < 0.05), but not *IL1B* (*P* < 0.329; Figure 5B). The findings support canonical regulation of these pain-relevant mRNAs by miR-133a-3p.

### Validation of miR-133a-3p transfection.

To confirm transfection efficiency, we measured miR-133a-3p expression after miR-133a-3p mimic or scramble transfection. Compared with scramble treatment, miR-133a-3p mimic treatment significantly increased miR-133a-3p expression (*P* < 0.001; Supplemental Figure 8A). Since miR-133a-3p shares a seed sequence with miR-1-3p, whose targets are well characterized (41), miR-1 was included as a positive control. As expected, the known miR-1 target *TWF1* was significantly downregulated in miR-1–transfected cultures (*P* < 0.001; Supplemental Figure 8B). These results confirm efficient transfection and provide additional support for the predicted miR-133a-3p binding partners.

### miR-133a-3p was present in neurons of the dorsal horn of the spinal cord and may alter binding partner RNA levels.

**Figure 6:**
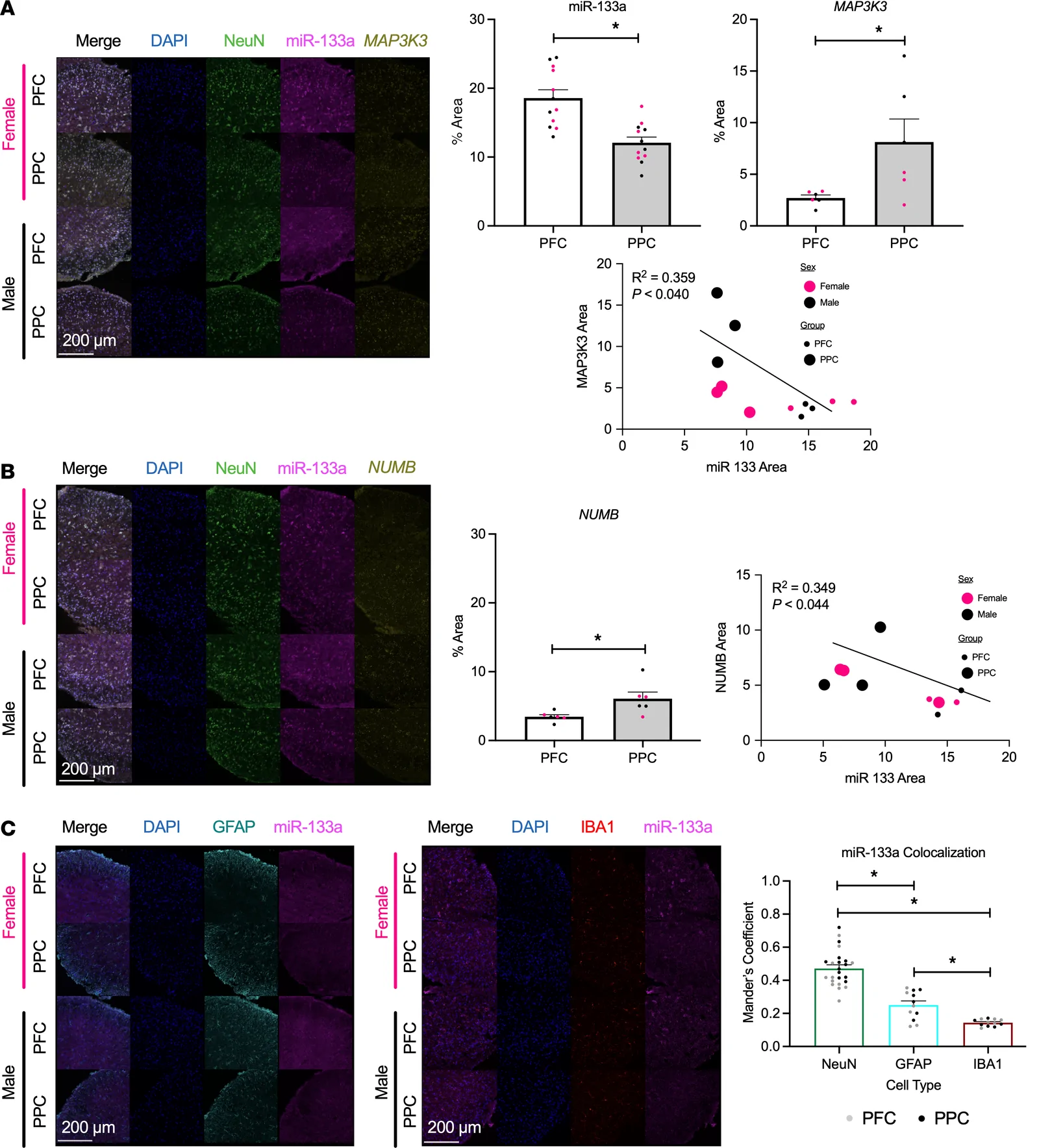
Neuronal miR-133a-3p regulates the levels of pain-relevant mRNA targets in vivo. (**A**) Representative confocal image of DAPI, NeuN, miR-133a-3p, and *MAP3K3* in the spinal dorsal horn of male and female PFC and PPC mice. Supporting graphs show quantification of signal intensity and Pearson’s correlation, which demonstrated significant negative relationships between miR-133a-3p and *MAP3K3*. (**B**) Representative confocal image of DAPI, NeuN, miR-133a-3p, and *NUMB* in the spinal dorsal horn of male and female PFC and PPC mice, with similar quantifications and findings. (**C**) Representative confocal images of DAPI, IBA1, GFAP, and miR-133a-3p in the spinal dorsal horn. Quantification of miR-133a-3p colocalization with neurons (NeuN^+^), astrocytes (GFAP^+^), and microglia (IBA1^+^) by Mander’s coefficient. *N* = 3–5 mice/sex/group; each data point represents the average of 3 sections per mouse. Scale bars: 200 μm. Data are presented as the mean + SEM. Data were analyzed by ANOVA followed by Tukey’s HSD post hoc test. *P < 0.05.

To test miRNA-mRNA relationships in vivo, we used a combinatory ISH and immunofluorescence (IF)/IHC approach to visualize RNA species alongside cell-type markers (42) in spinal cord and DRG tissue collected from male and female PFC and PPC mice on day 14. Compared with the PFCs, PPC mice had significantly lower levels of miR-133a-3p (*P* < 0.001) and greater levels of the predicted binding partners *MAP3K3* (*P* < 0.037) and *NUMB* (*P* < 0.025) in the spinal dorsal horn. *MAP3K3* and *NUMB* were significantly negatively correlated with miR-133a-3p in this region (*P* < 0.040 and *P* < 0.044, respectively; Figure 6, A and B). *HSP90B1* showed a nonsignificant negative correlation (*P* < 0.220), whereas *HSP90AA1* and *CRK* showed no clear relationship (Supplemental Figure 9, A–C).

*MAP3K3* levels were elevated in DRG of PPC mice (*P* < 0.030), despite no change in miR-133a-3p levels between groups (*P* < 0.922; Supplemental Figure 10A). *NUMB* levels were elevated only in male PPC mice (group × sex, *P* < 0.011, Tukey’s HSD post hoc, *P* < 0.008; Supplemental Figure 10B). *HSP90AA1* levels were also elevated (*P* < 0.012), whereas *HSP90B1* and *CRK* levels were unchanged (*P* < 0.652 and *P* < 0.813, respectively; Supplemental Figure 10, C–E). Together, the spinal cord *MAP3K3* and *NUMB* data support the RT-qPCR findings and the relationship between miR-133a-3p and these predicted targets. They also suggest that additional mechanisms contribute to regulation of these mRNAs.

Using the same approach, we examined miR-133a-3p localization in neurons (NeuN^+^), astrocytes (GFAP^+^), and microglia (IBA1^+^) within the spinal dorsal horn. miR-133a-3p was most abundant in neurons, followed by astrocytes and microglia, with all comparisons significantly different (all *P* < 0.05; Figure 6C). These findings support the adipocyte/EV/spinal cord axis hypothesis and identify dorsal horn neurons as the cell type most likely to be affected by adipocyte-derived miR-133a-3p loss following catecholamine activation.

### miR-133a-3p altered binding partner protein levels.

**Figure 7:**
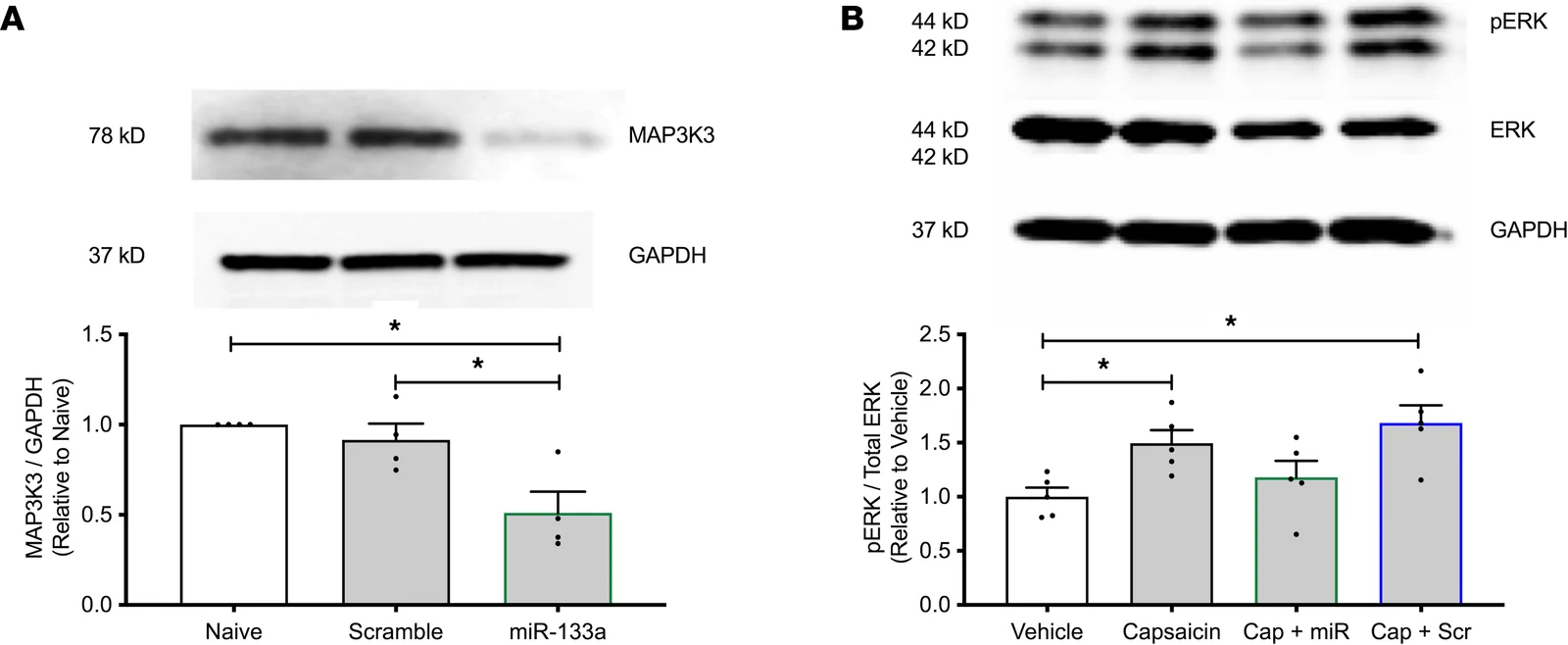
miR-133a-3p reduced MAP3K3 and inhibited ERK signaling in sensory neurons. (**A**) miR-133a-3p treatment for 48 hours reduced MAP3K3 protein levels. (**B**) miR-133a-3p treatment for 48 hours blocked capsaicin-induced pERK expression, a marker for nociceptor activity. *N* = 3–4 wells/group collected from 4–5 mice/group. Data are presented as the mean + SEM. Data were analyzed by ANOVA followed by a Tukey’s HSD post hoc test (**A**) and Dunnett’s multiple-comparison test (**B**). *P < 0.05.

To determine whether altered mRNA expression translated to altered protein expression, we quantified predicted binding partner proteins in primary neuron cultures treated with nothing (naive), scramble, or miR-133a-3p mimic. Compared with both naive and scramble groups, miR-133a-3p significantly reduced MAP3K3 protein levels (*P* < 0.008 and *P* < 0.021, respectively; Figure 7A). Despite altered mRNA expression in the RT-qPCR screen and luciferase assay, protein levels of CRK, HSP90AA1, HSP90B1, and NUMB were unchanged following miR-133a-3p treatment (Supplemental Figure 11). Together, these findings suggest that MAP3K3 protein expression is regulated by miR-133a-3p.

### miR-133a-3p reduced the phosphorylation of ERK in sensory neurons.

Sensory neurons increase ERK phosphorylation (pERK) relative to total ERK in response to noxious stimuli such as capsaicin, making pERK/ERK ratio a proxy for neuronal activity (43). Since MAP3K3 is an upstream kinase in the MAPK/ERK pathway, we predicted that miR-133a-3p would regulate nociception through this signaling cascade. In primary sensory neuron cultures, capsaicin increased the pERK/ERK ratio 1.49-fold relative to vehicle-treated controls. Pretreatment with a miR-133a-3p mimic prevented this increase (1.18-fold), whereas scramble treatment did not (1.68-fold). Capsaicin and capsaicin + scramble groups differed significantly from the vehicle controls (Dunnett’s post hoc, *P* < 0.05), while the miR-133a-3p group did not (Dunnett’s post hoc, *P* < 0.667), indicating that miR-133a-3p blocks capsaicin-induced pERK (Figure 7B).

### miR-133a-3p modulated the activity of sensory neurons in vitro.

**Figure 8:**
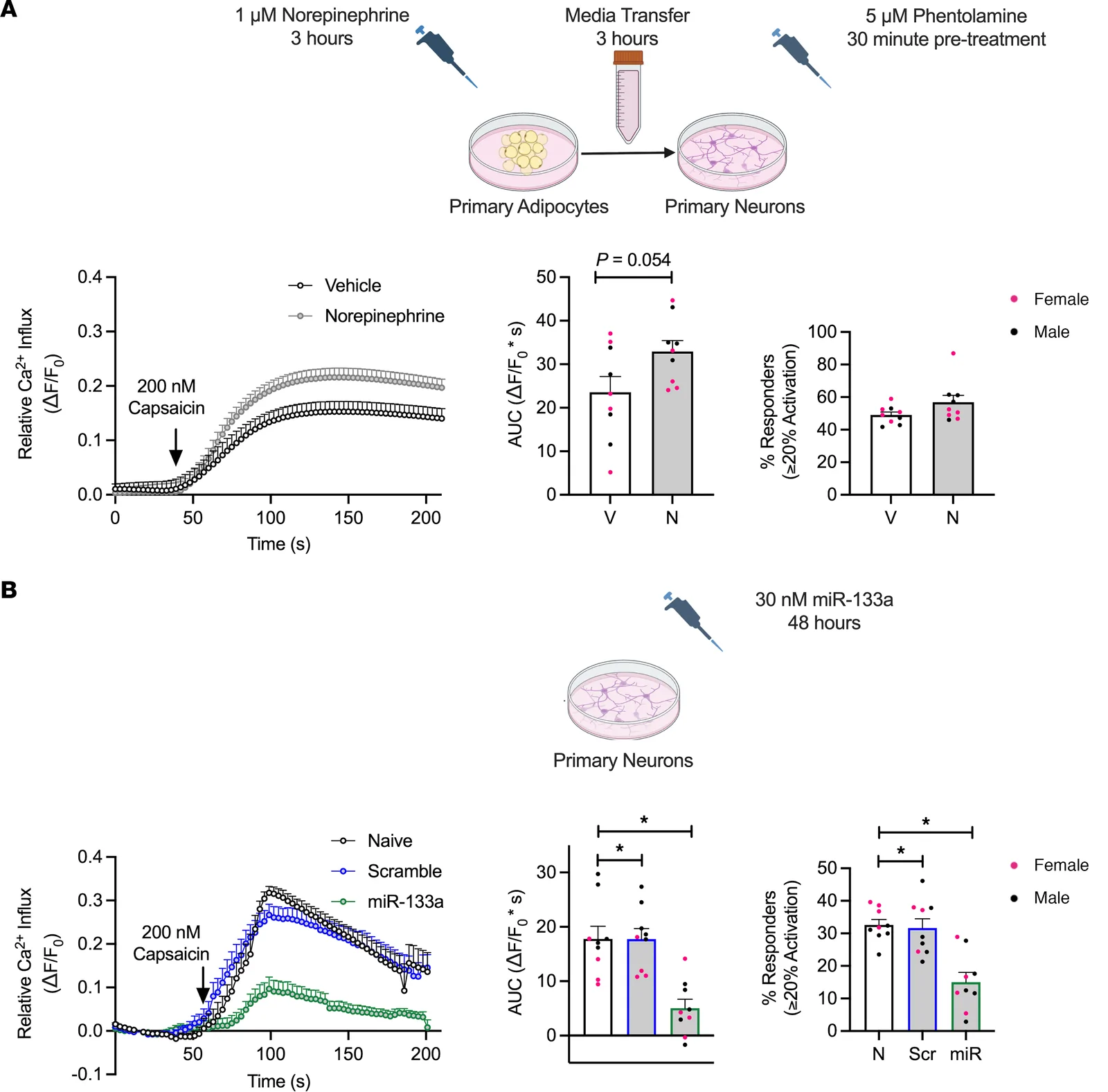
miR-133a-3p modulated sensory neuron activity in vitro. (**A**) Incubation of primary sensory neurons from Pirt-GCaMP3 mice with conditioned media from norepinephrine-treated adipocytes increased calcium influx in response to capsaicin challenge. *N* = 4–5 wells collected from 2 mice/sex/group. (**B**) miR-133a-3p treatment significantly reduced both relative calcium influx and the proportion of neurons reaching a maximum fluorescent peak ≥ 20% above baseline fluorescence during capsaicin challenge. *N* = 9 wells collected from 3 mice/sex/group. Data are presented as the mean + SEM. Data were analyzed by ANOVA followed by Tukey’s HSD post hoc test. *P < 0.05.

To test the hypothesis that miR-133a-3p in adipocyte-derived EVs regulates sensory neuron activity, we measured calcium signaling in primary neurons from Pirt-GCaMP3 mice using established methods (44). Primary adipocytes were treated with norepinephrine or vehicle for 3 hours, after which conditioned media containing secreted EVs was collected. This media was applied to Pirt-GCaMP3 neuron cultures pretreated with phentolamine for 30 minutes, followed by a 3-hour incubation to allow for EV uptake and downstream gene regulation. Neurons were subsequently challenged with capsaicin, and calcium influx was recorded. Compared with neurons incubated with vehicle-treated adipocyte media, neurons exposed to norepinephrine-treated adipocyte media exhibited greater overall calcium influx (*P* = 0.054; Figure 8A). These findings support the hypothesis that catecholamine activation of adipocytes reduces EV-associated miR-133a-3p, thereby increasing neuronal excitability.

To further establish a relationship between miR-133a-3p and nociception, separate Pirt-GCaMP3 neuron cultures were transfected with scramble or miR-133a-3p mimic. Compared with naive or scramble-treated neurons, miR-133a-3p–transfected neurons exhibited significant reductions in both overall calcium influx during capsaicin challenge (*P* < 0.001) and the proportion of neurons reaching the activation threshold during the 200-second recording period (*P* < 0.001; Figure 8B).

## Discussion

Here, we identified miR-133a-3p as a regulator of CPPCs across species, pain duration, and sex. We posit that catecholamine-driven downregulation of miR-133a-3p in white adipocytes promotes multisite mechanical hypersensitivity by regulating nociceptive mRNA targets. Using complementary in vitro and in vivo approaches in humans, mice, and rats, we tested the mechanism by which miR-133a-3p promotes analgesia. This work integrates adipose, stress, and miRNA contributions to pain processing and advances our understanding of CPPC etiology.

To our knowledge, this is the first study to demonstrate miR-133a-3p downregulation in circulating plasma as a signature of primary pain status across species and test this relationship mechanistically. In general, miR-133a-3p is highly expressed in myocytes and adipocytes, where it regulates differentiation and signaling processes (45, 46). In primary pain, we identified adipocytes as the major source of circulating miR-133a-3p. In response to norepinephrine, adipocytes reduce both local synthesis of, and EV-packaged secretion of, miR-133a-3p. This reduction may alter gene regulation at distant sites that normally receive adipocyte-derived miR-133a-3p. Consistent with this, treatment of neuronal or HEK293 cells with a miR-133a-3p mimic reduced expression and translation of pain-relevant genes such as *CRK*, *HSP90AA1*, *HSP90B1*, *MAP3K3*, and *NUMB*. Although these targets are implicated in pain biology (38, 44, 47–50) and immunology (51, 52), their relationship with miR-133a-3p in primary pain has not been investigated previously. In the spinal dorsal horn, miR-133a-3p was primarily localized to neurons and negatively correlated with both *MAP3K3* RNA and MAP3K3 protein. This association was predictive of primary pain status. Compared with PFC mice, PPC mice were more likely to exhibit lower miR-133a-3p levels and higher *MAP3K3* levels.

To directly assess the relationship between miR-133a-3p and mechanical hypersensitivity, we delivered an adipocyte-targeted viral vector designed to overexpress miR-133a-3p in PPC mice. Restoring miR-133a-3p expression not only reversed pain behaviors but also promoted analgesic responses above PFC baselines at paw, abdomen, and back sites. Together, these findings support a pathway whereby catecholamine stimulation of adipocytes reduces secretion of miR-133a-3p, leading to increased neuronal MAP3K3 expression, pERK, and nociception. This pathway promotes multisite hypersensitivity.

CPPCs have well-established genetic risk factors, including loss-of-function *COMT* variants (5–7), that may interact with environmental factors, such as stress (7, 53), mood disorders (54), bone density (55), and BMI (56, 57). Together, these factors suggest convergence of adipose and sympathetic nervous system signaling in pain regulation. During muscle injury, adipose can facilitate the infiltration of regenerative cells critical to healing (58), likely through extensive genetic remodeling. miRNAs are well suited to coordinate such responses because a single miRNA can regulate hundreds of genes (17). Circulating miRNAs have been previously associated with skeletal muscle damage (45, 46) and individual CPPCs (59–62), including miR-133a-3p (63). However, shared miRNA signatures across CPPCs, their mechanistic roles, and their links to adipose biology have remained largely unexplored.

Adipose tissue receives sympathetic input from somatosensory neurons (64, 65). Under basal conditions, white adipocytes synthesize and secrete miR-133a-3p and other miRNAs into circulation via EVs (66). However, catecholamine stress signaling alters both EV abundance and cargo composition, including miRNAs (67–69). These adipocyte-derived EV miRNAs regulate gene expression in distant tissues (22) and are implicated in metabolic disorders (70, 71) and nervous system dysfunction (72). One study demonstrated that adipose-derived EV miR-9-3p may contribute to cognitive dysfunction in the hippocampus of mice with type 2 diabetes mellitus (73). Our findings extend these principles to primary pain.

The strength of this study lies in targeted approaches alongside extensive controls. First, direct manipulation of miR-133a-3p levels in vitro and in vivo allowed us to test its role in mechanical hypersensitivity. Second, rather than relying on in silico target prediction (74), we used 4 complementary approaches (RT-qPCR, luciferase assay, combinatory ISH/IF/IHC, and Western blot) to experimentally validate miRNA-mRNA relationships. Third, the use of 3 species (human, rat, and mouse) increases the generalizability and translational relevance of our findings.

Despite these strengths, several limitations remain that we plan to address in future work. First, the receptor regulating miR-133a-3p downregulation has not been definitively identified. Consistent with prior work, our data indirectly support ADRB3 because norepinephrine reduced miR-133a-3p expression in adipocytes but not DRG neurons, matching the known distribution of ADRB3 expression (75, 76). Yet, selective pharmacologic or genetic tools are needed for direct confirmation. Second, EV experiments were performed in vitro. Although adipocyte-conditioned media directly influenced nociceptor activity, future studies should investigate EV transfer and function in vivo. Third, although multiple approaches supported a relationship between miR-133a-3p and *MAP3K3*, additional experiments such as coprecipitation assays or direct manipulation of *MAP3K3* expression would further strengthen the proposed mechanism.

In conclusion, miR-133a-3p homeostasis is critical to the development of primary pain, with reduced levels driving mechanical hypersensitivity at multiple body sites. This effect likely results from increased levels of pain-relevant genes, including *MAP3K3*, following reduced delivery of adipocyte-derived EV miR-133a-3p to the spinal cord. These findings identify a previously unrecognized adipocyte/neuron signaling pathway in primary pain and support miR-133a-3p as a potential therapeutic target for CPPCs.

## Methods

### Sex as a biological variable.

All experiments measured sex or gender as a factor, with 3 exceptions. (a) Females were chosen for the small RNA sequencing study as CPPCs are more prevalent in females than in males (77). (b) The VBD cohort collected samples only from women. (c) Though the FMS cohort included 6 males, it was not powered to detect sex differences. In the absence of a significant main effect or interaction including sex or gender, data were collapsed.

### Animal model of primary pain.

Animals had ad libitum access to standard laboratory chow and water and were housed in plastic cages with wood shavings in a temperature-controlled facility on a 12-hour light/dark cycle with lights on at 7 am. Adult female Sprague-Dawley rats (*N* = 20) were bred in-house, weighed 200–400 g, and were housed 2/cage at the University of North Carolina at Chapel Hill (UNC). Adult C57BL/6 mice of both sexes (*N* = 100) were either bred in-house on a Jackson Laboratory background or ordered directly from The Jackson Laboratory, weighed 16–24 g, and were housed 2–5/cage at Duke University.

As published (12, 38), the animal model of primary pain combined COMT inhibition with OR486 (Tocris Bioscience) with 3 days of repeated swim stress. The acute time point (3–6 hours) utilized a single i.p. injection of 30 mg/kg of OR486. Sustained time points (over 14 days) utilized continuous s.c. delivery of 15 mg/kg/day of OR486 for 14 days via a surgically implanted osmotic minipump (model 2002, Durect Corporation). The small RNA-seq experiment utilized peripheral delivery of 1.67 mg/kg/day of the ADRB2 antagonist ICI-118,511 and the ADRB3 antagonist SR59230A in a second minipump with a catheter terminating in the hind limb. OR486, ICI-118,511, and SR59230A were dissolved in a 5:3:2 ratio of DMSO (DMSOStore), 0.9% saline (Cytiva), and absolute ethanol (Thermo Fisher Scientific) (9).

### Surgical procedures.

For implantation of osmotic minipumps in rats, a sterile technique was employed throughout the duration of all procedures according to IACUC requirements. Animals were anesthetized by isoflurane inhalation (Covetrus). A small incision was made over the shoulder blades, and pumps were implanted. Stainless steel wound clips (Braintree Scientific) were used to close the wounds. For tissue collection in mice, animals were deeply anesthetized with isoflurane in a non-contact gas chamber and perfused intracardially with 0.1 M PBS. For Western blot and RT-qPCR studies, tissues were isolated, collected, flash-frozen on dry ice, and stored at –80°C. For IHC analysis, PBS perfusion was followed by 4% paraformaldehyde (PFA) perfusion in PBS. After 12 hours of postfixation in 4% PFA, adipose and muscle tissue were paraffin-embedded, while spinal cord and DRG were dehydrated in 30% sucrose solution and embedded in OCT compound. Samples collected from the model include whole blood, WAT, brown adipose tissue, DRG, spinal cord, and muscle.

### RNA-seq.

Whole blood was collected from rats in PAXgene Blood RNA tubes (BD Biosciences). RNA was isolated from blood using the PAXgene blood miRNA kit (Qiagen). The quantity and quality of the RNA were measured using a NanoDrop 1000 (NanoDrop Technologies) and an Agilent 2100 Bioanalyzer (Agilent Technologies), respectively. RNase-free water was used to normalize all samples to approximately 250 ng/μL. RNA samples were then shipped to the Next-Generation Sequencing and Bioinformatics Core at the University of Texas Health Science Center at San Antonio for small RNA-seq using the Illumina HiSeq 2000 with the TriLink Small RNA Library Prep Kit. Samples were measured with 3 technical replicates.

### Clinical cohorts.

As described (21), women with VBD (*N* = 45) and PFCs (*N* = 22) were recruited to the UNC Pelvic Pain Clinic. Exclusion criteria included (a) age < 21 or > 45; (b) breastfeeding, pregnant, or menopause at time of participation; (c) medical conditions such as thyroid disorder, diabetes, or epilepsy; and (d) confirmed diagnosis of a comorbid urogenital condition such as interstitial cystitis. Full demographic data can be found in our published work (21).

As described (78), individuals with FMS (*N* = 24) and PFCs (*N* = 24) were recruited to the Alan Edwards Pain Management Unit of the Montreal General Hospital. Exclusion criteria included (a) inability to provide informed consent in English or French, (b) age <40, (c) a diagnosis of a different pain condition that would explain pain, (d) an uncontrolled medical or psychiatric condition, and (e) prior or current history of drug abuse, including alcohol. Full demographic data can be found in Supplemental Table 3.

### RT-qPCR.

The TaqMan system (Thermo Fisher Scientific) was used for mRNA quantification, while LNA miRNA PCR assays (Qiagen) were used for miRNA quantification. Refer to Supplemental Table 4 for specific kit and probe catalog numbers. Total RNA was isolated using TRIzol reagent (Thermo Fisher Scientific). RNA was transcribed to cDNA using either 2 μg (mRNA) or 5 μg (miRNA) of nuclease-free H_2_O (Sigma-Aldrich). In the miRNA procedure, cel-miR-39-3p and UniSp6 templates were added to the reverse transcription mix to serve as an exogenous control. Exogenous controls were selected because no consistent control miRNA was known across the tissue types measured and to control for reverse transcription efficiency. RT-qPCR reactions were performed in triplicate on a QuantStudio 5 Real-Time PCR System with Design Studio Software v5.1.2 (Thermo Fisher Scientific). Ct values were normalized using the 2^–ΔΔCt^ method (79), with *18S* and *ACTB* as reference genes in mRNA analysis and cel-miR-39-3p and UniSp6 in miRNA analysis.

### Cell culture.

All cells were maintained in a 5% CO_2_ incubator at 37°C with complete media replacement every 2 days. For experiments utilizing primary differentiated adipocyte and neuron culture from WT C57BL/6 mice, standard methods were employed (44). Briefly, aseptically removed tissues were incubated with collagenase (40 mg/mL) and dispase-II (200 mg/mL) (Both Roche) diluted in HBSS (Thermo Fisher Scientific) at 37°C for 120 minutes. The homogenate was passed through a 70 μm mesh filter into a fresh 50 mL conical tube. Samples were centrifuged at 150 × *g* for 10 minutes at 4°C, and pelleted cells were resuspended in culture media. Preadipocytes were grown to 95% confluency with DMEM/F12/GlutaMAX-I media (Thermo Fisher Scientific) supplemented with 10% FBS and 1× penicillin-streptomycin. After reaching confluency, preadipocytes were differentiated into mature white adipocytes for 4 days by adding 0.5 mM IBMX, 1 μM rosiglitazone, 5 μM dexamethasone, and 0.5 μg/mL insulin to the base medium. Mature adipocytes were then maintained for 4 days with base medium supplemented with 0.5 μg/mL insulin. DRGs were seeded on glass coverslips and grown in base media supplemented with 2% B27 (Thermo Fisher Scientific).

For experiments utilizing HEK293T cells (MilliporeSigma), cells were grown with DMEM + GlutaMAX-I media supplemented with 10% FBS and 1× penicillin-streptomycin. All experiments were performed at passage ≤ 10.

### Catecholamine activation.

Each well of mature white adipocytes or neurons was treated with 1 μM norepinephrine (Sigma-Aldrich) in distilled H_2_O or vehicle for 3 hours. After media removal, cells were washed twice with 2 mL DPBS (Thermo Fisher Scientific), collected in 0.5 mL TRIzol reagent (Thermo Fisher Scientific), and incubated overnight at 4°C.

### EV isolation and particle analysis.

Recovered media was passed through a 100 kDa Amicon filter (MilliporeSigma) and centrifuged at 3,500*g* at 4°C for 30 minutes in a swing bucket rotor. Size-exclusion chromatography was performed with qEV columns (Izon) to isolate EVs. Isolated vesicles underwent several downstream analyses. For miRNA quantification, total RNA was extracted from vesicles with the Total Exosome RNA & Protein Isolation Kit (Thermo Fisher Scientific).

For nanoparticle tracking analysis, adipocyte-derived EV size distribution and particle number were measured with a Zetaview instrument (Particle Metrix). Purified adipocyte EVs were diluted 1:25 in PBS before analysis. For accuracy, only EVs ≥ 45 nm were analyzed.

For transmission electron microscopy (EM), 900 μL of purified EVs pooled from 3 samples per group was concentrated down to 20 μL using 100 kDa MWCO centrifugal filters and fixed with 80 μL of 4% PFA (ACROS Organics) overnight. 10 μL of the fixed solution was loaded onto a Formvar/carbon-coated EM grid (200 μm Cu). Following a 10-minute, room temperature incubation and a distilled H_2_O wash, grids were stained with 2% uranyl acetate before EM image acquisition on a JEOL JEM-1400Plus electron microscope.

### AAV delivery.

Mice received an i.v. tail vein injection of a targeted recombinant AAV vector containing either miR-133a-3p or a nonsense sequence on day 7 of the PPC model. rAAV8-mAP2.2-pre-miR-133a-3p-eGFP and rAAV8-mAP2.2-nonsense-eGFP were synthesized from the pAAV-mir-GFP backbone (Abcam). Abcam provided in vitro transduction quality control. When coupled with rAAV8, murine adiponectin (mAP2.2) is a promoter restricted to subcutaneous and visceral fat depots (80). A titer of 2 × 10^11^ delivered by i.v. tail vein injection 2 weeks before effective adipose transduction was selected based on published results in male mice 9–13 weeks old (81). Tissue transduction was validated by a presence/absence test (Ct < 36) of eGFP and relative quantification of miR-133a by RT-qPCR.

### Mechanical allodynia and hyperalgesia.

Before baseline assessment, mice were handled and habituated for 3 days in stainless steel mesh testing chambers positioned on a wire mesh table for 1 hour/day. On test days, mice were habituated to the testing chambers for 30 minutes prior to testing. For plantar sites, mechanical allodynia was assessed by applying von Frey filaments (0.008–4 g; Stoelting Co.) to the hind paw plantar surface using the up-down method to calculate 50% paw withdrawal threshold (82–84). Mechanical hyperalgesia was determined by measuring response frequency to a suprathreshold 0.4 g filament applied 10 times (83). For abdominal and back sites, animals were shaved 1–2 days before baseline pinprick testing. For the back, a 1 × 1cm^2^ area above the posterior superior iliac spine was marked. For the abdomen, a 1 × 1cm^2^ area surrounding the umbilicus was marked. The inner needle of a spinal needle (25GX3.00N, BD Biosciences) was applied to each site, and the number and type of responses to 15 stimulations were recorded (85). Response categories included normal/innocuous (simple withdrawal), noxious (elevating the area for extended periods of time, flinching, and licking), and null responses (no withdrawal).

### Motor function assessment.

Mice were randomly assigned to 2 groups. Motor function was assessed at baseline and day 24 using a Rotarod apparatus (IITC Life Science). Each assessment consisted of 3 consecutive trials separated by 10-minute intertrial intervals, during which the rotation speed gradually increased from 4 to 40 rpm over a 5-minute period. The latency to fall was recorded for each trial and averaged for analysis.

### Dual-luciferase assay.

Dual-luciferase constructs (*Gaussia* luciferase [GLuc] and secreted alkaline phosphatase [SEAP]; pEZX-MT05) containing the full-length WT 3′UTR of genes of interest were purchased from GeneCopoeia. HEK293T cells at 70%–80% confluency in 96-well plates were transfected with 50 ng construct using Lipofectamine 3000 (Thermo Fisher Scientific). 5 hours later, 30 nM miR-133a-3p mimic or scramble control (Thermo Fisher Scientific) was cotransfected into the same wells using INTERFERin (PolyPlus)in serum-free media. Media (50 μL/well) was collected 48 hours after cotransfection and stored at –20°C until use. For the assay, 10 μL of media was mixed with 100 μL of either GLuc or SEAP substrate buffer according to the Secrete-Pair Dual Luminescence Assay Kit (GeneCopoeia) protocol. Luminescence was measured using an Infinite M200 Pro and i-Control software v1.10 (Tecan Group). Individual samples were run in triplicate and measured twice. The average GLuc/SEAP ratio of each scramble control group was used to normalize relative expression differences between groups.

### IF and IHC.

Standard methods of fluorescent or colorimetric protein visualization by IF or IHC were used for paraffin-embedded and non-paraffin-embedded, PFA-fixed tissues (86). Briefly, tissues were sliced with an HM 355S apotome (Thermo Fisher Scientific) or CryoStar NX50 cryostat (Thermo Fisher Scientific) and mounted on Superfrost Plus microscope slides (Thermo Fisher Scientific). Paraffin-embedded tissues were dewaxed with fresh xylene (Sigma-Aldrich) and underwent antigen retrieval with 1× citrate buffer (Sigma-Aldrich). For stains requiring peroxidase, slides were incubated with 3% H_2_O_2_ (Sigma-Aldrich) for 60 minutes. All slides were then washed with PBS for 3 × 5 minutes. Blocking was performed with 10% normal donkey serum (NDS; Jackson ImmunoResearch) in PBS for 1 hour. Primary antibodies were diluted in 5% NDS and used to incubate sections for 12 hours at 4°C. Slides were washed for 3 × 15 minutes in fresh PBS with 0.1% Tween 20 (PBST; Thermo Fisher Scientific). Secondary antibodies were diluted in 5% NDS, incubated for 1 hour, and washed for 3 × 15 minutes in fresh PBST. For antibodies requiring amplification, the TSA Plus Cyanine 3 System (Akoya Biosciences) manufacturer protocol was followed. For costaining, when required, additional peroxidase activity was quenched after the final stain by incubating sections with 1× TrueBlack Lipofuscin autofluorescence quencher (Biotium) for 1 minute. Slides were rinsed with PBS and sealed with glass coverslips (VWR) using glycerol mounting medium with DAPI (Abcam). All antibodies and kit components are listed in Supplemental Table 5.

### Western blotting.

Standard methods of protein detection by immunoblotting were used for cell and tissue lysates (87). Briefly, cells were lysed in RIPA buffer (Sigma-Aldrich) and tissues in T-PER tissue protein extraction reagent (Thermo Fisher Scientific) supplemented with protease and phosphatase inhibitors (Sigma-Aldrich). Lysates were cleared by centrifugation at 11,000 × *g* for 15 minutes. Protein concentration was determined using a BCA assay (Thermo Fisher Scientific). Equal amounts of protein were mixed with 4× Laemmli sample buffer (Bio-Rad) containing β-mercaptoethanol (Sigma-Aldrich) and denatured at 100°C for 5 minutes. Samples were loaded onto SDS-PAGE gels (Bio-Rad) and separated at 100 V until the bromophenol blue dye front ran out of the gel. Proteins were transferred to PVDF membranes using an iBlot 2 Dry Blotting System (Thermo Fisher Scientific). Membranes were rinsed in TBST (Bio-Rad) and blocked with 5% nonfat dry milk (Bio-Rad) in TBST for 1 hour at room temperature. Primary antibodies were diluted in 3% BSA and incubated with membranes overnight at 4°C. Membranes were then washed 3 × 10 minutes in TBST and incubated with HRP-conjugated rabbit or mouse secondary antibodies (Cell Signaling Technology) diluted in blocking buffer for 1 hour at room temperature. Following incubation, membranes were washed 3 × 10 minutes in fresh TBST. Bands were visualized with the SuperSignal West Pico PLUS Chemiluminescent Substrate (Sigma-Aldrich) on a ChemiDoc MP Imaging System (Bio-Rad) and quantified using ImageJ (NIH). Relative target protein levels were normalized to GAPDH expression for each sample and averaged per mouse (*N* = 3–4 wells per mouse, 4–5 mice per group). The vehicle control group average was used to normalize relative expression differences between groups. When necessary, membranes were stripped using stripping buffer (Thermo Fisher Scientific) and reprobed. All antibodies and reagents are listed in Supplemental Table 5.

### Confocal microscopy.

Images were captured at ×20 magnification using a Zeiss 980 confocal microscope. For AAV validation images, acquisition settings were as follows: 405 nm (0.2% power, 750 V gain, 6.33 s frame time), 488 nm (1% power, 750 V gain, 6.33 s frame time), and 594 nm (3% power, 750 V gain, 6.33 s frame time), collected with 5 *Z*-stack slices at 4 μm intervals. For ISH with IF/IHC images, acquisition settings were as follows: 405 nm (5% power, 655 V gain, 1.89 s frame time), 488 nm (7% power, 650 V gain, 1.89 s frame time), 561 nm (5% power, 650 V gain, 1.89 s frame time), and 639 nm (8% power, 745 V gain, 1.89 s frame time), collected with 13 *Z*-stack slices at 9.6 μm intervals.

### RNAscope ISH standard.

Using published methods of RNAscope ISH combined with IHC (42), colorimetric probes targeting miR-133a-3p and mRNA transcripts were hybridized. Briefly, sections underwent progressive dehydration (50%, 70%, 100% ethanol), H_2_O_2_, and protease IV treatment. Probes diluted in dilutant buffer were added to sections for 2 hours at 40°C to hybridize. Signal was amplified sequentially using Amp1, Amp2, and Amp3 reagents with wash buffer rinses between steps. HRP-C1 was applied to the sections for 15 minutes at 40°C, followed by washing. Cy3 and/or Cy5 fluorescent dye reagent in TSA buffer was then added before a final wash. ISH staining was performed prior to IHC. All antibodies and kit components are listed in Supplemental Table 5.

### ISH and IHC quantification.

Images were quantified in 1 of 2 ways: signal intensity or signal colocalization using FIJI v. 2.14.0/1.54f. Signal intensity was determined 1 channel at a time using percent area coverage of signal from an average intensity *Z*-projected image in adjust threshold mode. Signal colocalization was determined by comparing 2 channels at a time using the coloc2 plug-in to quantify Mander’s coefficient of correlation and co-occurrence (88). Additional analyses included a standard Pearson’s correlation between miRNA and mRNA target signal intensity.

### In vitro calcium imaging.

Using established methods (38), primary neuron activity from Pirt-GCaMP3 mice grown on glass coverslips was assessed in 2 calcium imaging experiments. In the first experiment, primary adipocytes were stimulated with norepinephrine or vehicle for 3 hours. During the final 30 minutes, neuron cultures were treated with or without 5 μM phentolamine (Sigma-Aldrich). Phentolamine pretreatment was necessary to block inhibition from residual norepinephrine acting through α receptors and unmask the excitatory properties of conditioned adipocyte media (Supplemental Figure 12) (89, 90). Adipocyte media was then used to completely replace the neuron culture media for 3 hours before imaging. Calcium influx was measured every 3 seconds using a ×20 lens at an excitation wavelength of 488 nm. Baseline recordings were collected for 60 seconds in imaging buffer (pH 7.4) containing CaCl_2_ (2 mM), NaCl (140 mM), KCl (5 mM), MgCl_2_ (1 mM), HEPES (10 mM), and d-(+)-glucose (10 mM). Capsaicin (200 nM) in imaging buffer was applied for 141 seconds. Relative calcium influx was calculated as ΔF/F_0_, the ratio of change in fluorescence intensity (F_t_–F_0_) to baseline (F_0_). Percent responders was calculated as the percentage of neurons that had at least a 20% increase in fluorescence from baseline during the capsaicin challenge.

In the second experiment, no primary adipocytes were used. Only primary neurons were transfected with 30 nM miR-133a mimic or scramble control for 48 hours prior to imaging.

### Statistics.

All graphs representing statistical analyses were created using GraphPad Prism 11 (GraphPad Software). For small RNA-seq of rat samples, miRNA expression was quantified using a previously described small RNA-seq read mapping pipeline (91). This pipeline, which utilizes the Bowtie (92) and SHRiMP (93) software packages, allows for identification of both established and putative miRNAs that may act as regulatory hubs in disease. All miRNAs with Benjamini-Hochberg–adjusted *P* < 0.05 are reported in this study.

For non-bioinformatic experiments, statistical analyses within each experiment began with a single omnibus ANOVA appropriate for the experimental design (e.g., 1-, 2-, or 3-way ANOVA) to minimize type I error. When significant interactions were observed, data were further analyzed using lower-order ANOVAs and appropriate post hoc testing. Repeated-measures ANOVA was used for longitudinal behavioral assessments. Tukey’s HSD or Dunnett’s multiple-comparison test was applied when necessary. In the absence of significant interactions, only main treatment effects were evaluated. Experiments containing a single factor and 2 groups were analyzed using a 2-tailed Student’s *t* test. *P* < 0.05 was considered statistically significant. When necessary, *P* values reported in Results were adjusted for multiple comparisons (Tukey’s test unless otherwise specified).

For some experiments, data transformation was justified. In RT-qPCR, OpenArray, and dual-luciferase experiments, fold change values were log transformed to balance under- and overexpression. In mechanical hyperalgesia assays, noxious responses were converted to a percentage of total responses.

### Study approval.

All rodent work conformed to the NIH Guide for the Care and Use of Laboratory Animals (94) and the Animal Research: Reporting of In Vivo Experiments guidelines (95). Rodents were selected because nociceptive pathways and behaviors are well characterized in these species, and validated pain behavior assays are widely available (96–98). All rat procedures were approved by the IACUC at UNC. All mice procedures were approved by the IACUC at Duke University. The VBD study was approved by the UNC IRB, and the FMS study was approved by the McGill University IRB.

### Data availability.

Values for all data points in graphs are reported in the Supporting Data Values file. Data are presented as mean ± SD, except for RNA-seq data. The RNA-seq data have been deposited in NCBI’s Gene Expression Omnibus under accession number GSE338452. Full, uncropped Western blots (Figure 7, A and B, and Supplemental Figure 10) are available in the supplemental materials.

## Author contributions

NPH designed research studies, conducted experiments, analyzed data, and wrote the manuscript. JC, YW, YQ, XZ, BPC, XG, YLP, and M Kanke conducted experiments. MEK, CC, and PS designed research studies. M Karaky, CBM, FM, and LD provided reagents and clinical samples. AGN designed research studies, analyzed data, and edited the manuscript.

## Conflict of interest

The authors have declared that no conflict of interest exists.

## Funding support

This work is the result of NIH funding, in whole or in part, and is subject to the NIH Public Access Policy. Through acceptance of this federal funding, the NIH has been given a right to make the work publicly available in PubMed Central.

- American Heart Association grant 23IPA1054013 (to CC).
- NIH/National Institute of Biomedical Imaging and Bioengineering grant 1R21EB035738 (to CC).
- Diabetes Research Center, Washington University, NIH/National Institute of Diabetes and Digestive and Kidney Diseases grant P30DK020579 (to CC).
- Professorship in Pain Research from Pfizer Canada (to LD).
- Canadian Excellence Research Chairs grant CERC09 (to LD).
- Canada Research Chair (Tier 2) (to LD).
- NIH/National Institute of Neurological Disorders and Stroke (NINDS) grants R03NS106166, R01NS109541, and P01NS045685 (to AGN).
- NIH/NINDS grant F31NS130861 (to NPH).
- NIH/National Institute of General Medical Sciences grant T32GM133352 (to NPH).

## Supplementary Material

Supplemental data

Unedited blot and gel images

Supporting data values

## Version 1 07/21/2026

In-Press Preview

## Version 2 09/01/2026

Electronic publication
