# Supplementary material for: Catecholamine-mediated release of miR-133a-3p from adipocytes regulates the onset of chronic primary pain
Source: J Clin Invest. 2026 Jul 21;136(17):e197345. doi: 10.1172/JCI197345 (PMC13528929; doi:10.1172/JCI197345)
Supplement: Supplementary file 1 [file jci-136-197345-s020.pdf]

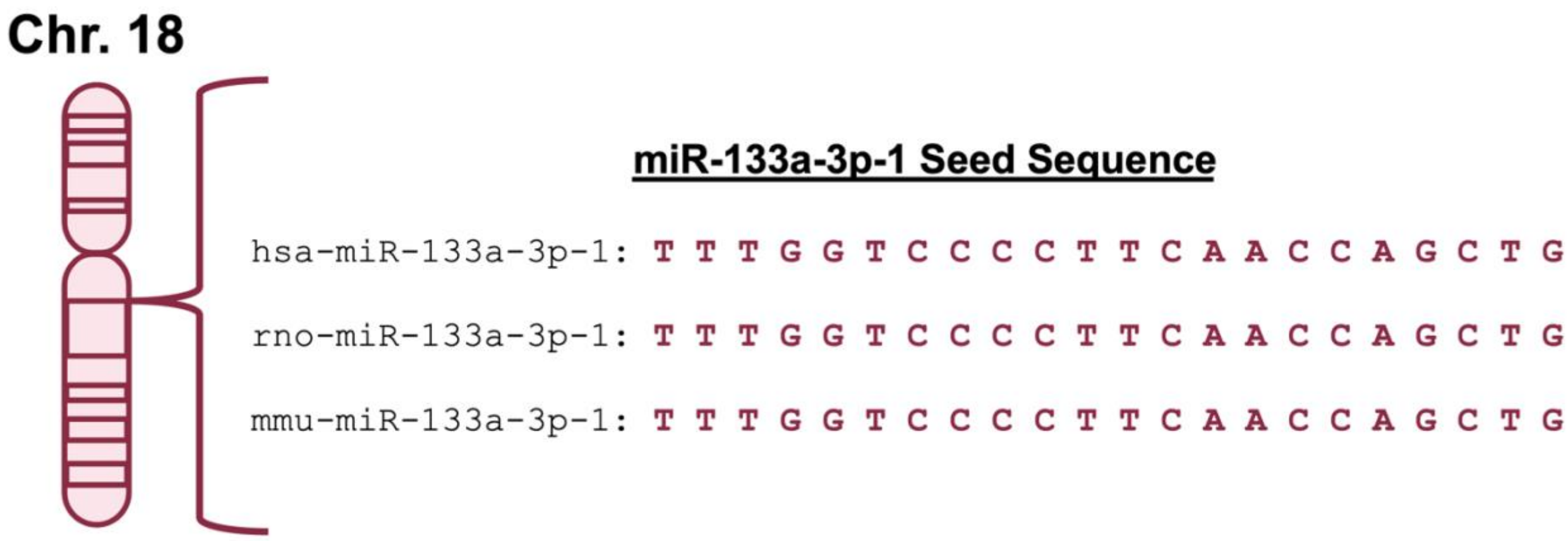

Supplemental Figure 1. Homology of miR-133a-3p. Conserved 22-nucleotide sequence of miR-133a-3p-1 across *Homo sapiens* (hsa; Human), *Rattus norvegicus* (rno; Rat), and *Mus musculus* (mmu; Mouse). miR-133a-3p-1 is located on chromosome 18, while the identical duplicate miR-133a-3p-2 is found on chromosome 20.

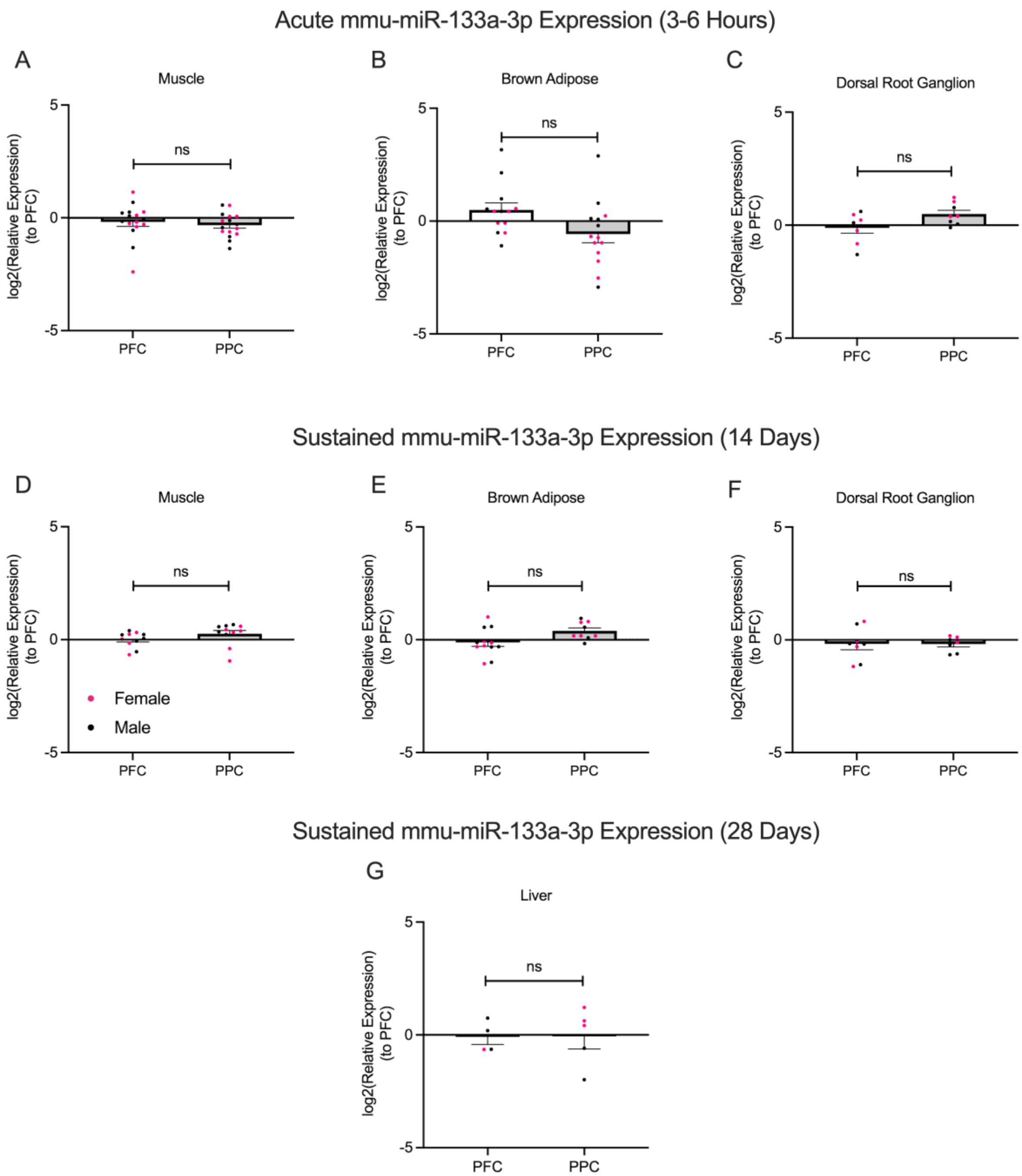

Supplemental Figure 2. miR-133a-3p expression was unchanged in muscle, brown adipose tissue, DRG, and liver. Compared to PFC mice, PPC mice exhibited no differences in miR-133a-3p expression at acute (3-6 hours) or sustained (14 or 28 days) time point in **(A,D)** muscle, **(B,E)** brown adipose tissue, (**C,F)** DRG, or **(G)** liver. Data are mean + SEM. Data were analyzed by ANOVA followed by Tukey's HSD post-hoc test. $*P < 0.05$.

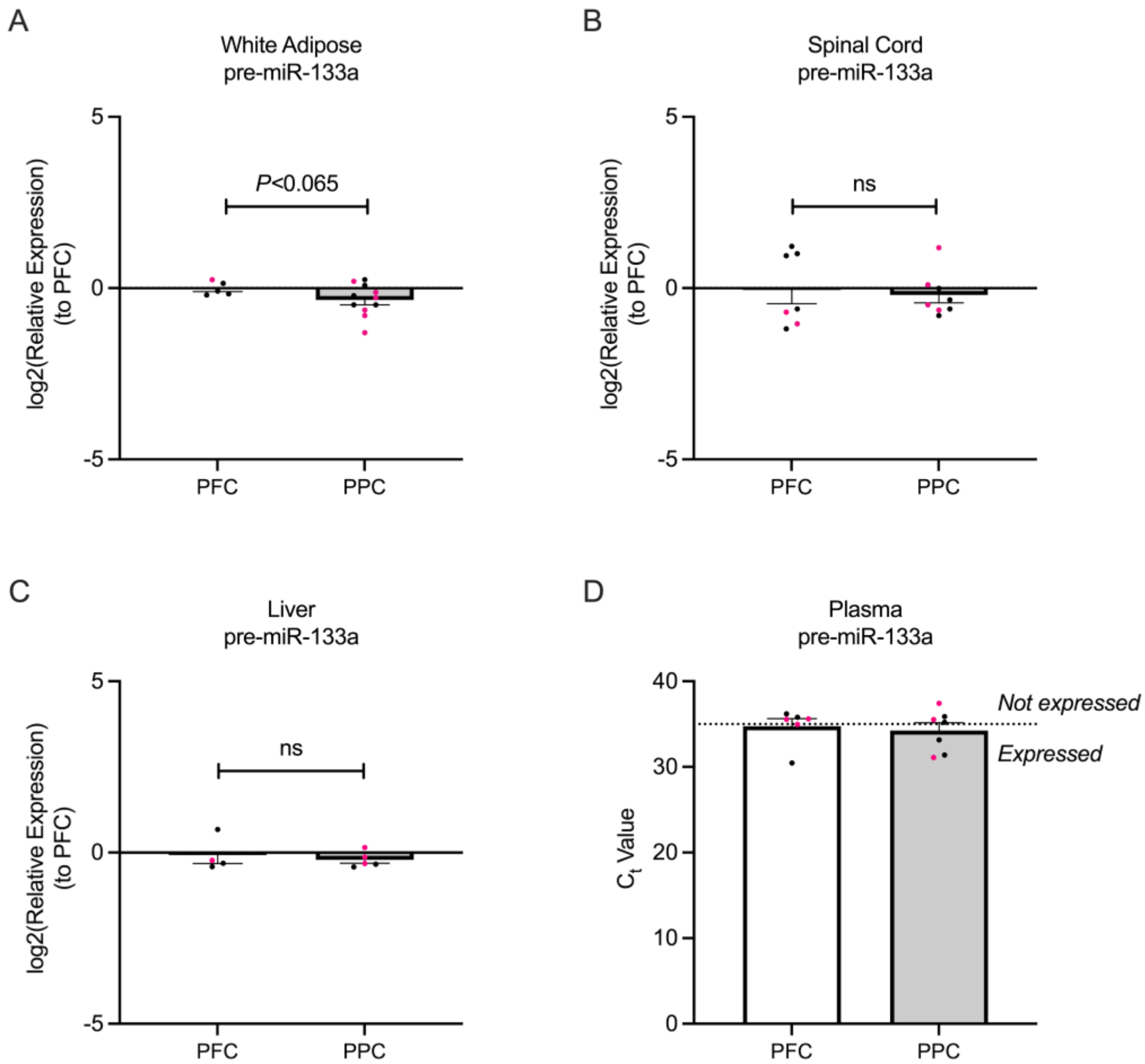

Supplemental Figure 3. Pre-miR-133a-3p expression across tissues at Day 28. Compared to PFC mice, PPC mice exhibited a trend towards reduced pre-miR-133a-3p expression in **(A)** white adipose tissue, but not in **(B)** spinal cord or **(C)** liver. **(D)** Pre-miR-133a-3p was not detected in plasma. Data are mean + SEM/ Data were analyzed by ANOVA followed by Tukey's HSD post-hoc test. **P* < 0.05.

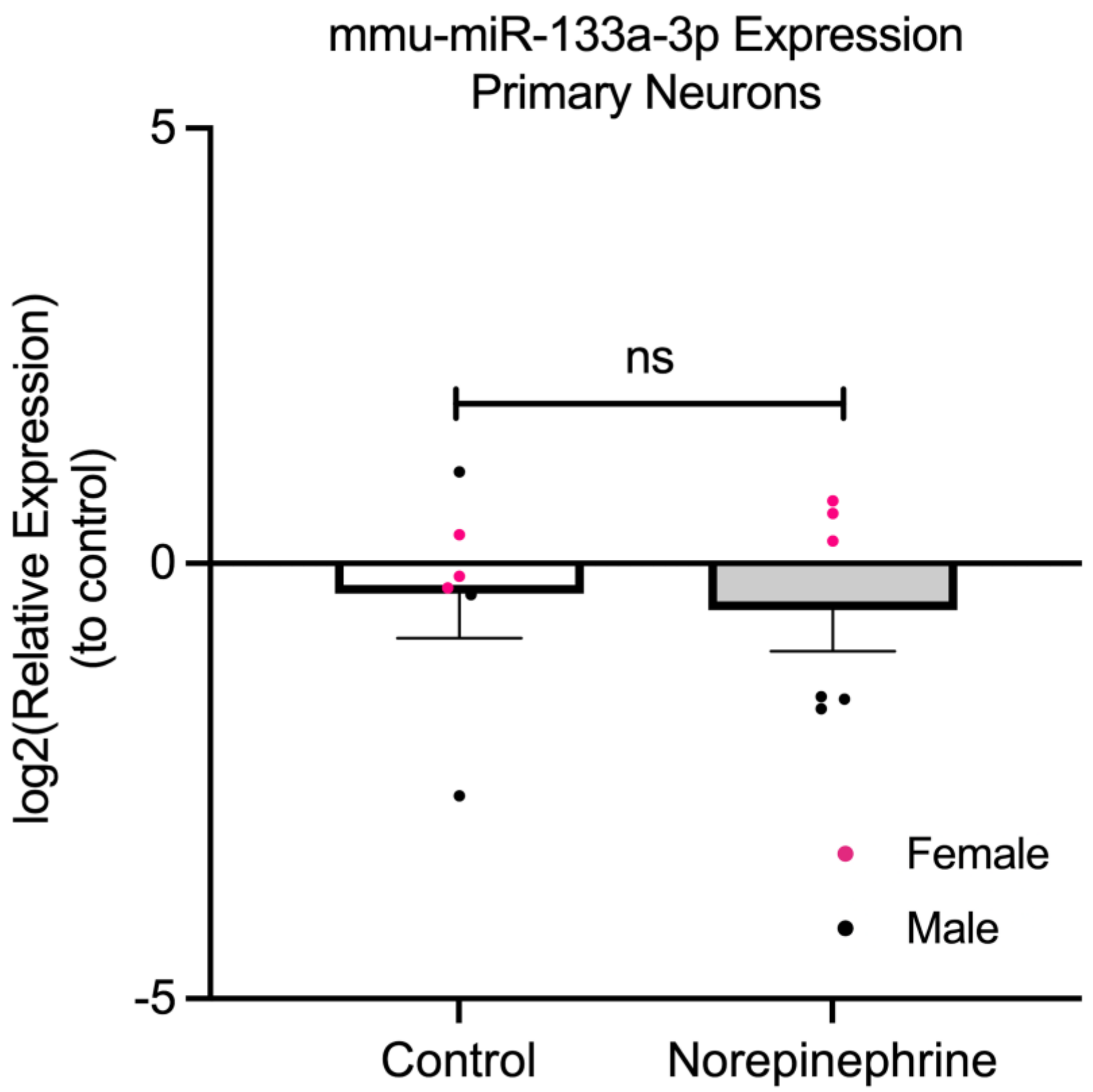

Supplemental Figure 4. Norepinephrine did not alter miR-133a-3p expression in primary neurons. (A) Compared to vehicle treatment, norepinephrine treatment did not alter miR-133a-3p expression in primary neurons. N=3 wells/sex/group. Data are mean + SEM. Data were analyzed by ANOVA followed by Tukey's HSD post-hoc test. *$P < 0.05$.

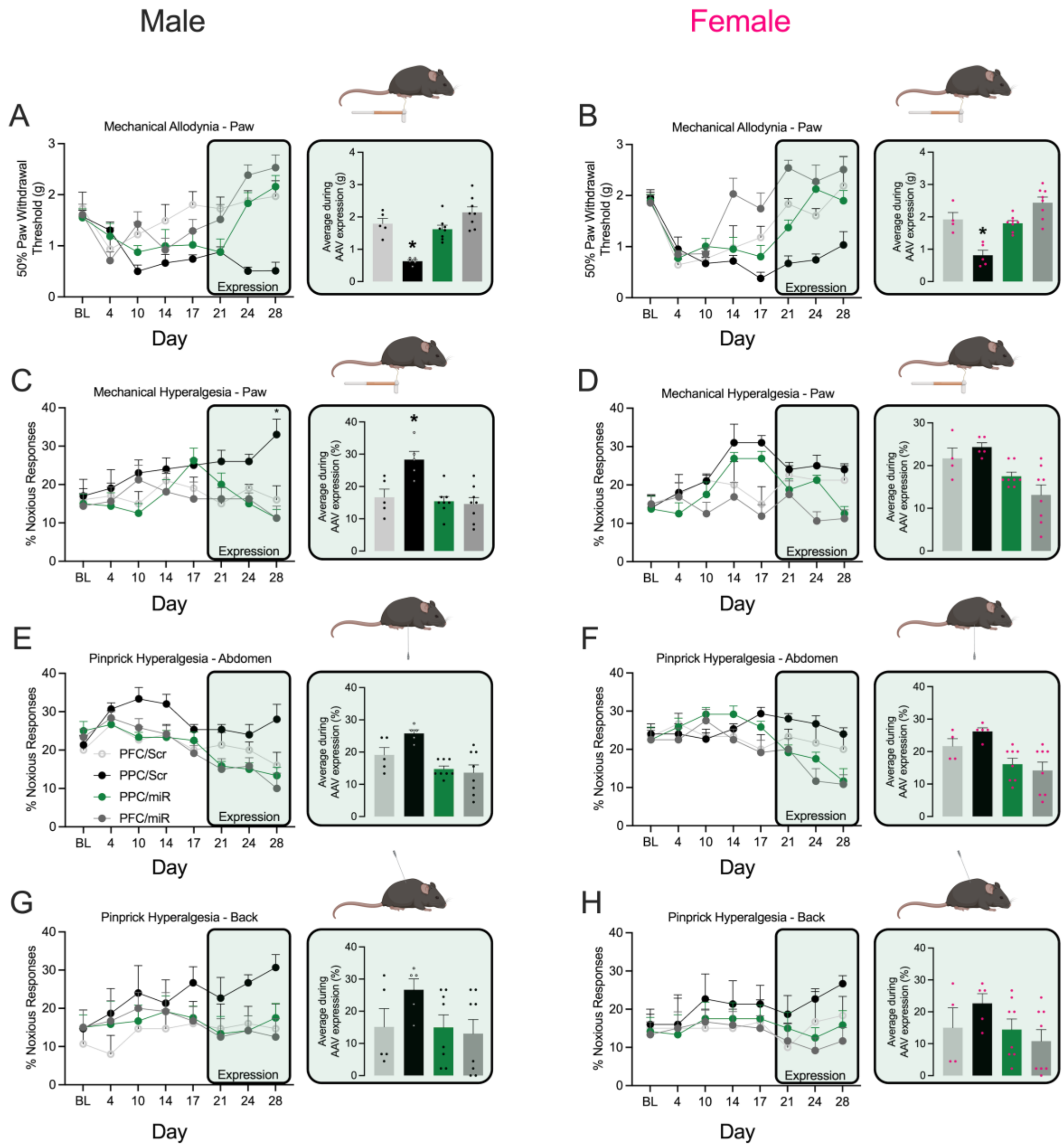

Supplemental Figure 5. Adipose-specific overexpression of miR-133a-3p reversed multi-site mechanical hypersensitivity in both sexes of a rodent model of primary pain. Data from Figure 4 are shown separated by sex. **(A-H)** Compared to PFC/Scramble mice, PFC/miR-133a-3p mice exhibited increased tolerance to mechanical stimuli in the paw and abdomen. Compared to PPC/Scramble, PPC/miR-133a-3p mice exhibited reversal of mechanical hypersensitivity during active vector expression (Days 21-28). During this period, PPC/miR-133a--3p mice were statistically indistinguishable from PFC/Scramble controls. N=3-8 mice/sex/group. Data are mean + SEM. Data were analyzed by mixed-effects model with repeated measures and Dunnett's multiple-comparisons test. Bar graph callouts were analyzed by three-way ANOVA followed by Dunnett's multiple-comparisons test. **P* < 0.05 to PFC/Scramble at matched time points.

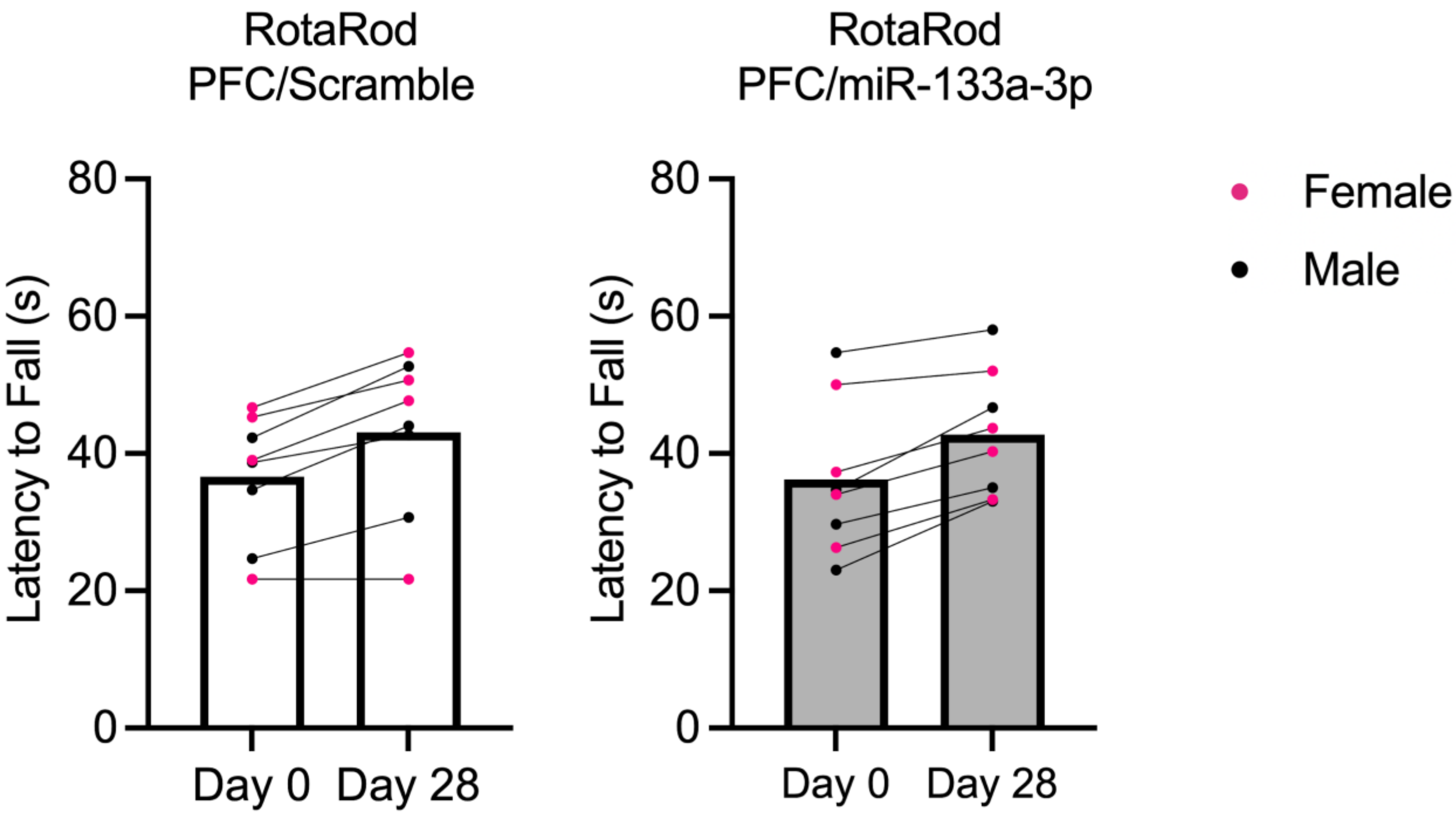

Supplemental Figure 6. Adipose-specific overexpression of miR-133a-3p did not alter motor function. Compared to PFC/Scramble mice, PFC/miR-133a-3p mice exhibited no differences in latency to fall at baseline or Day 28 post-AAV tail-vein injection. N=3-4 mice/sex/group. Data are mean + SEM. Data were analyzed by ANOVA with repeated measures followed by Tukey's HSD post-hoc test.

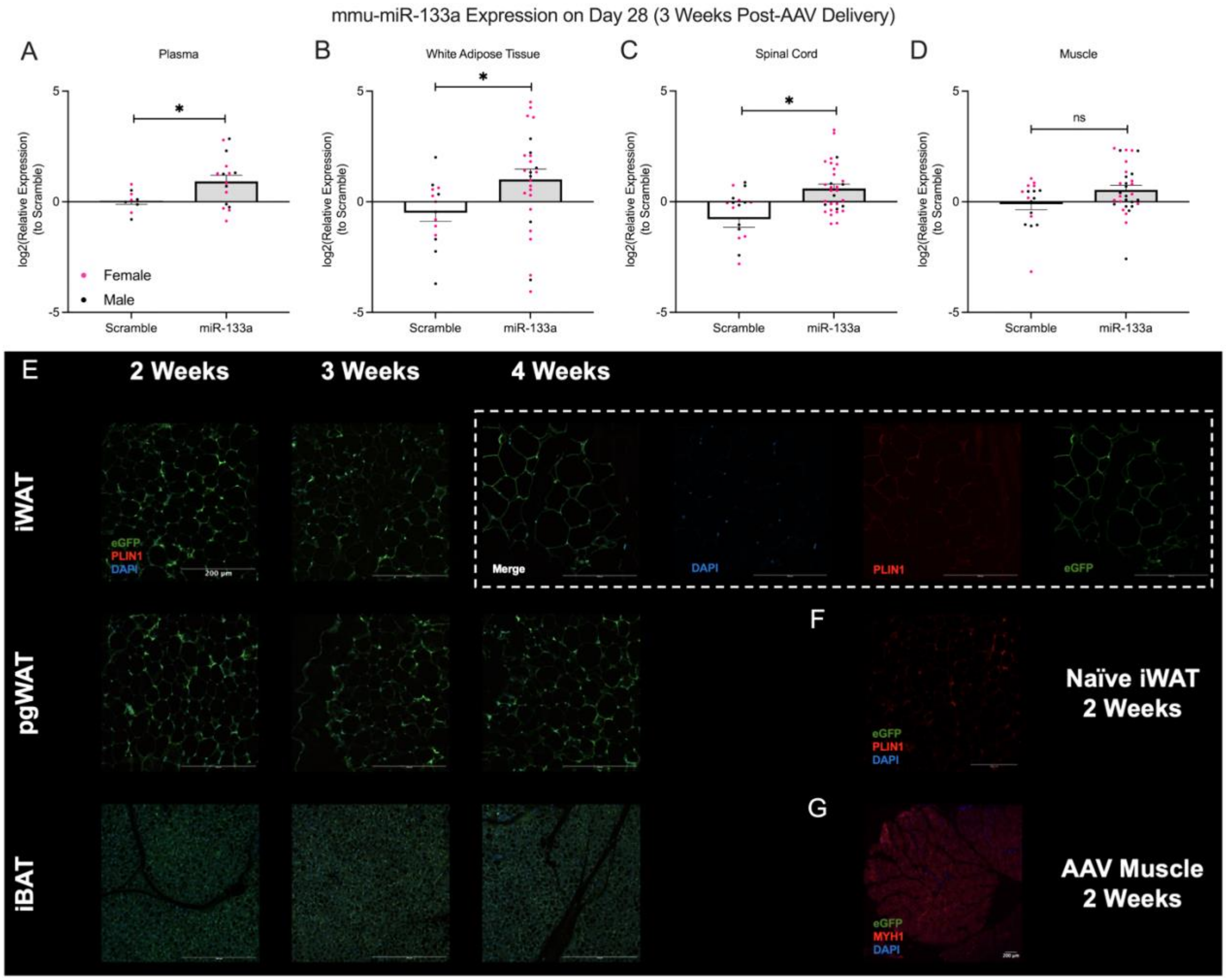

Supplemental Figure 7. Intravenous administration of rAAV8-mAP2.2-miR-133a-3p-eGFP drove adipose-specific expression as early as 2 weeks post-administration. 3 weeks after miR-133a-3p vector delivery (Day 28 in model), miR-133a-3p levers were significantly greater in **(A)** plasma, **(B)** white adipose tissue, and **(C)** spinal cord, but not in **(D)** muscle. **(E)** Using IF/IHC, eGFP was detected 2, 3, and 4 weeks after vector delivery in inguinal white (iWAT), perigonadal white (pgWAT), and intrascapular brown adipose tissue (iBAT). eGFP was not detected in either **(F)** adipose tissue from saline-treated mice, or **(G)** muscle from mice given the miR-133a-3p vector. N=4-9 mice/sex/group. Panels E-G show representative images from the same cohort. Scale bar = 200 μm. Data are mean + SEM. Panels A-D were analyzed by ANOVA followed by Tukey's HSD post-hoc test. *$P < 0.05$. Abbreviations: PLIN1 (Perilipin 1; adipocyte marker), MYH1 (Myosin heavy chain type I; muscle marker).

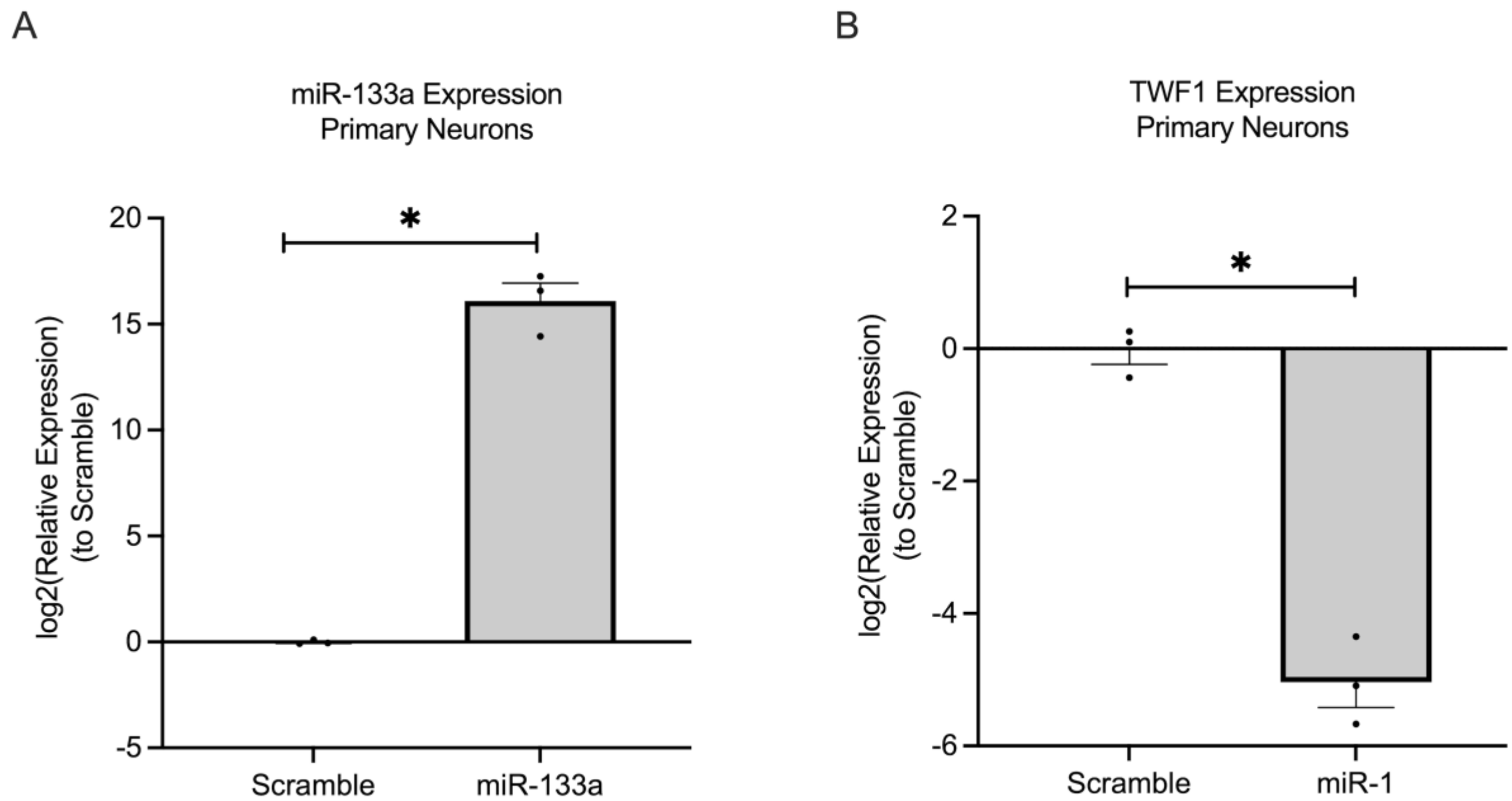

Supplemental Figure 8. Efficient miRNA transfection in primary neurons (A) miR-133a-3p transfection significantly increased miR-133a-3p expression in primary neurons. **(B)** miR-1-3p transfection significantly decreased expression of TWF1, a known miR-1-3p target, in primary neurons. Data are mean + SEM. Data were analyzed by ANVA followed by Tukey's HSD post-hoc test. *$P < 0.05$.

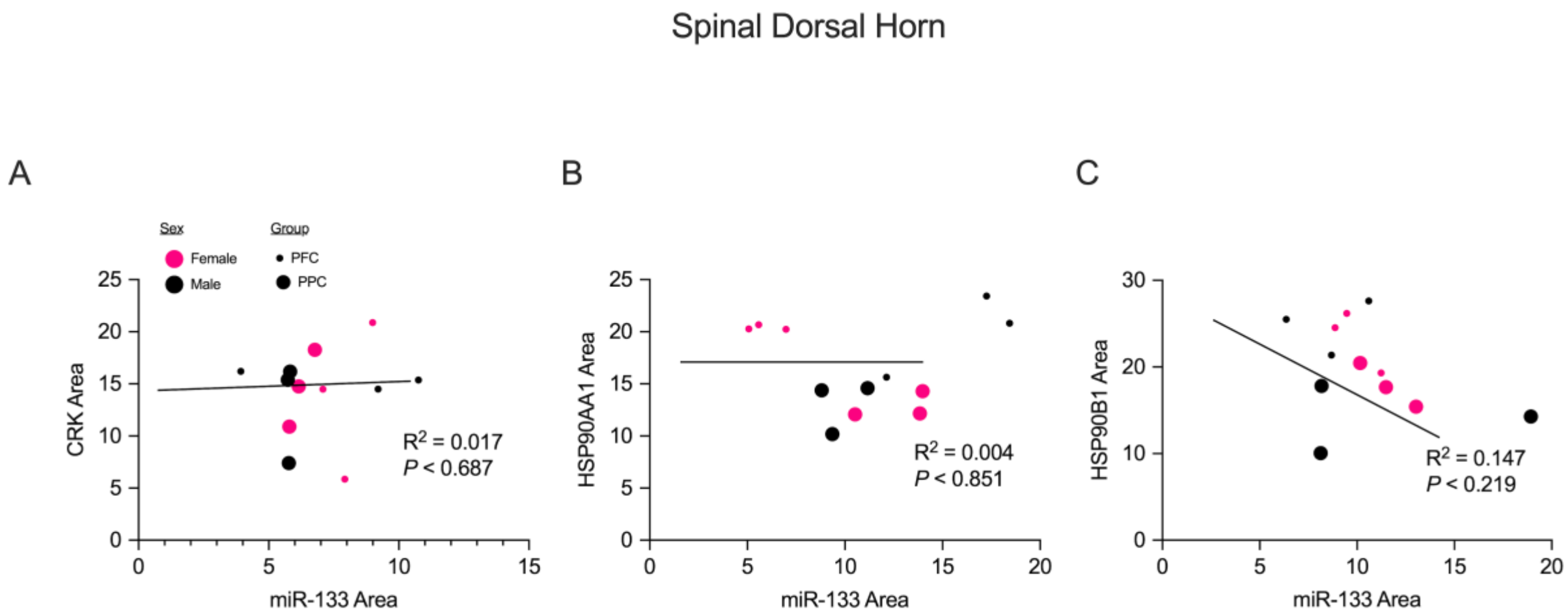

Supplemental Figure 9. ***in vivo*** **levels of miR-133a-3p and predicted targets in spinal dorsal horn.** As in Figure 6B, Pearson's correlations between miR-133a-3p and the predicted binding partners **(A)** *CRK*, **(B)** *HSP90AA1*, and **(C)** *HSP90B1* are shown. N=3-5 mice/sex/group; each data point represents the average of 3 sections per mouse. Data were analyzed by linear regression.

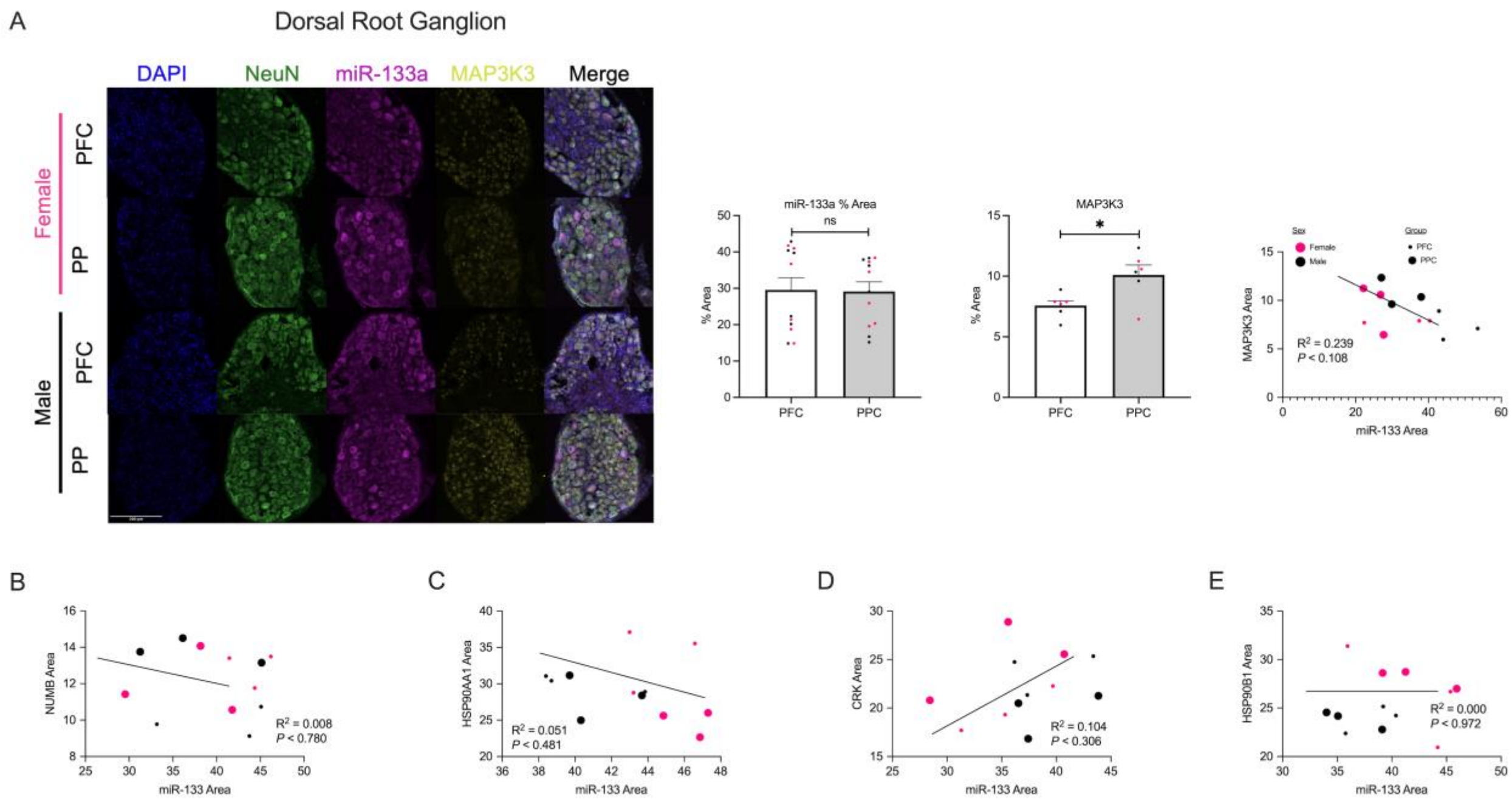

Supplemental Figure 10. *in vivo* levels of miR-133a-3p and predicted targets in L4-L5 DRG. (A) Representative confocal microscopy images of DAPI, NeuN, miR-133a-3p, and *MAP3K3* expression in L4-L5 DRG from PFC and PPC mice. Quantifications of signal intensity and their Pearson's correlation show unchanged levels of miR-133a-3p, but significantly increased levels of *MAP3K3.* No significant differences were observed for **(B)** *NUMB*, **(C)** *HSP90AA*, **(D)** *CRK*, or **(E)** *HSP90B1*. N=3-5 mice/sex/group; each data point represents the average of 3 sections per mouse. Data are mean + SEM. Scale bar=200 µm. Panel A was analyzed by ANOVA followed by Tukey's HSD post-hoc test and linear regression. Panels B-E were analyzed by linear regression. *$P < 0.05$.

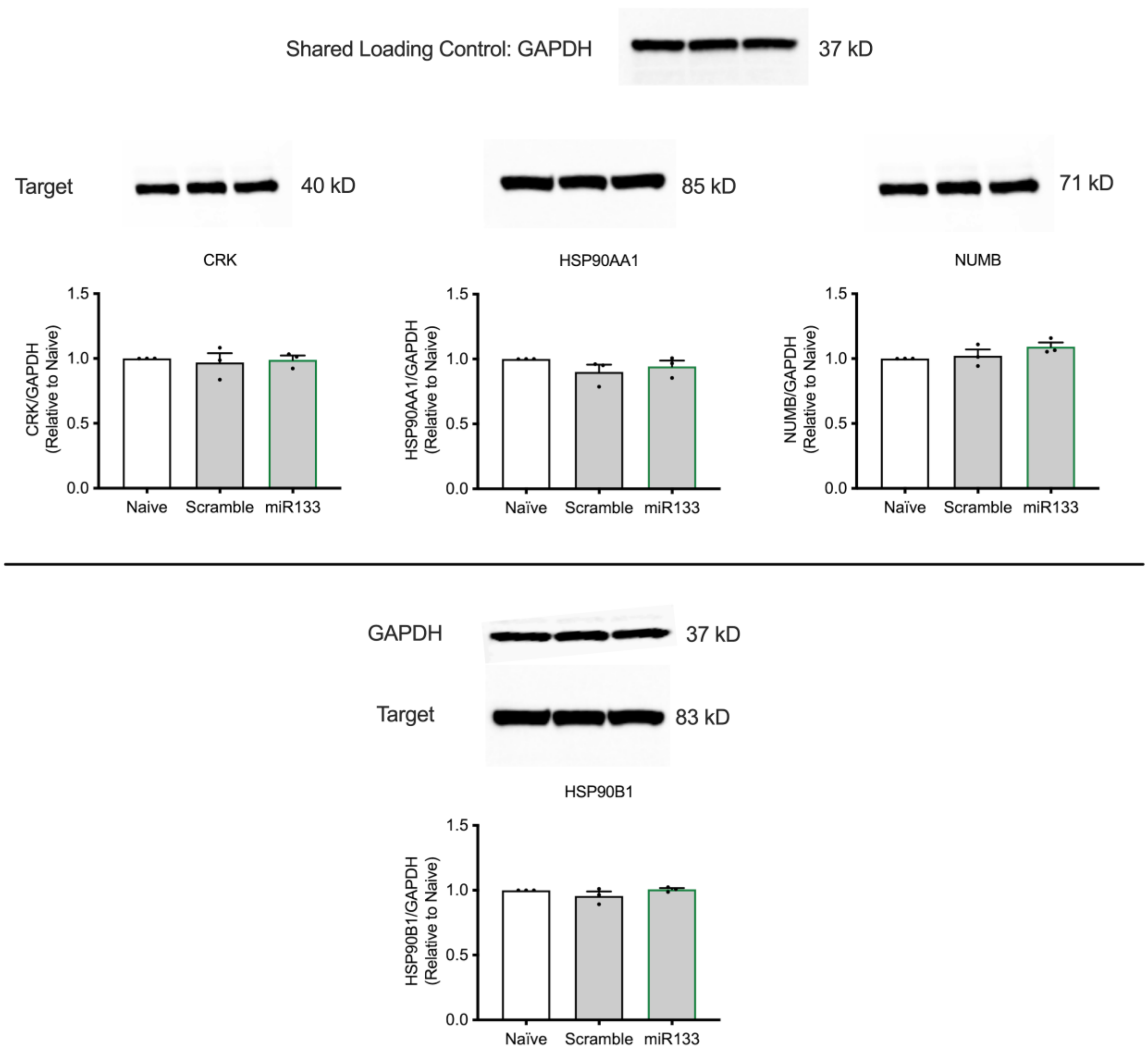

Supplemental Figure 11. Protein levels of predicted miR-133a-3p binding partners. Unlike MAP3K3, transfection of primary neurons with a miR-133a-3p mimic did not significantly alter protein expression of CRK, HSP90AA1, HSP90B1, or NUMB, compared to naive- or scramble-treated neurons. N=3-4 wells from 4-5 mice/group. Data are mean + SEM. Data were analyzed by ANOVA followed by Tukey's HSD post-hoc test.

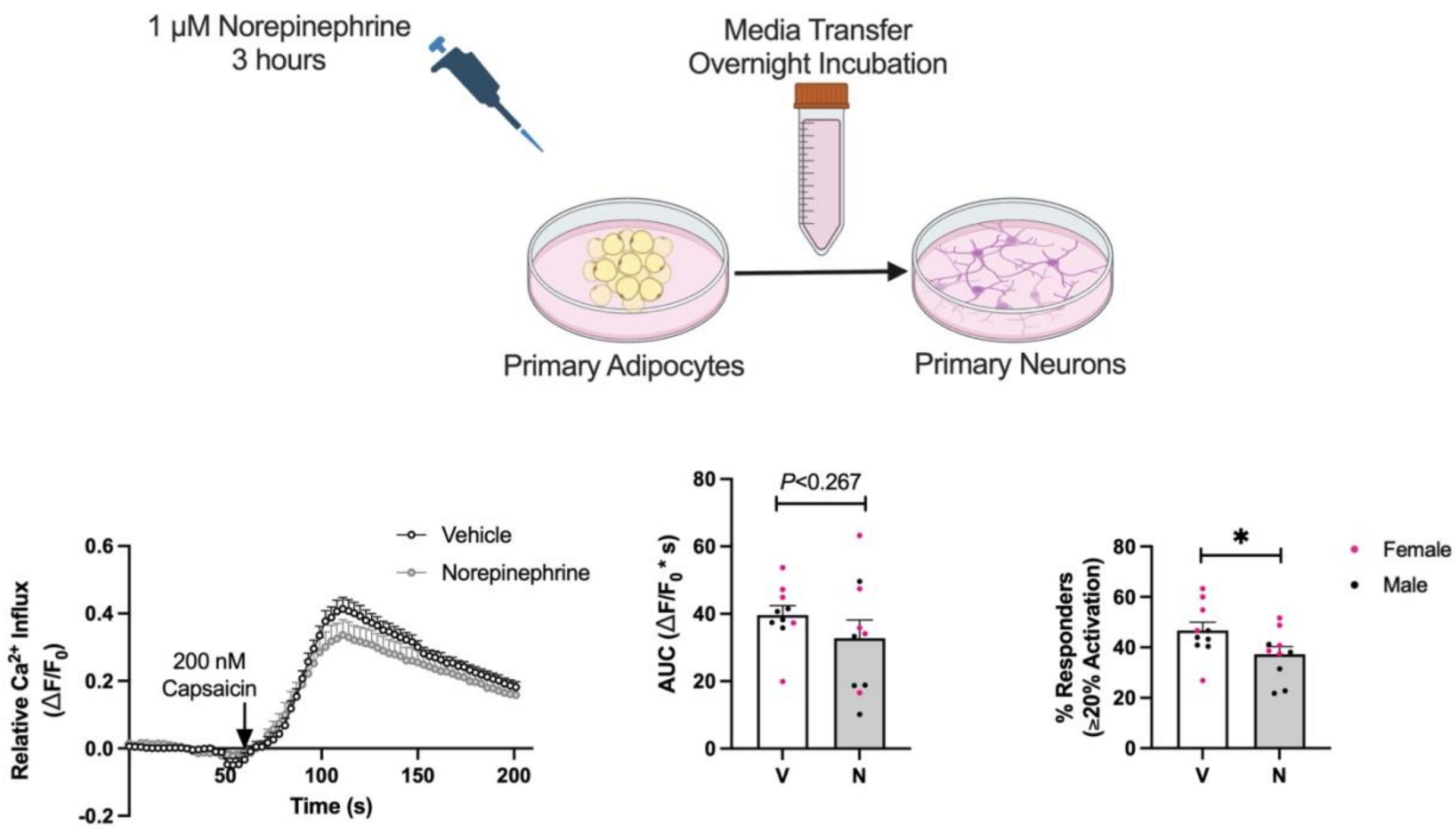

Supplemental Figure 12. Residual norepinephrine in conditioned media suppressed neuron activity. Without phentolamine pretreatment, fewer neurons incubated with norepinephrine-treated adipocyte media respond to a capsaicin challenge. N=5 wells/sex/group. Data are mean + SEM. Data were analyzed by ANOVA followed by Tukey's HSD post-hoc test. *$P < 0.05$.

Table 1. Percent of samples (N=13/group) where eGFP was detected on day 28.

Tissue	% eGFP Present ($C_T \leq 36$)	% eGFP Absent ($C_T > 36$ or Undetermined)
Subcutaneous WAT	88	12
Visceral WAT	96	4
Spinal Cord	8	92
Muscle	4	96

Table 2. STarMIR binding probability of miR-133a-3p and mRNA targets.

Target Name (NCBI Accession)	Best Probability	Best Location	Probability of 3'UTR-Seed	Probability of 3'UTR-Seedless
CRK-II (NM_133656.5)	0.896	CDS-Seedless	0.000	0.576
HSP90AA1 (NM_010480.5)	0.882	CDS-Seedless	0.600	0.829
HSP90B1 (NM_011631.1)	0.835	CDS-Seedless	0.000	0.726
MAP3K3 (NM_011947.4)	0.783	CDS-Seedless	0.000	0.617
NUMB (NM_001136075.2)	0.969	5'UTR-Seedless	0.000	0.779

Table 3. FMS Cohort Demographics

	Pain-free Controls N=24	FMS N=24	P-value
Age, N (%)			0.06
40-50	8 (33.33%)	5 (20.83%)	
51-60	8 (33.33%)	16 (66.67%)	
61-74	8 (33.33%)	3 (12.50%)	
Mean (SD)	56.00 (8.15)	54.00 (7.36)	
Gender, N (%)			0.70
Women	19 (79.17%)	23 (87.50%)	
Men	5 (20.83%)	1 (4.17%)	
Race/Ethnicity, N (%)			0.16
White	17 (70.83%)	21 (70.90%)	
Black	0 (0.00%)	0 (0.00%)	
Arab	0 (0.00%)	0 (0.00%)	
Latino/Hispanic	1 (4.17%)	2 (8.33%)	
Filipino	0 (0.00%)	0 (0.00%)	
South Asian	0 (0.00%)	0 (0.00%)	
West Asian	2 (8.33%)	0 (0.00%)	
East Asian	0 (0.00%)	0 (0.00%)	
Southeast Asian	1 (4.17%)	0 (0.00%)	
Jewish	1 (4.17%)	0 (0.00%)	
Aboriginal	0 (0.00%)	1 (4.17%)	
Other	1 (4.17%)	0 (0.00%)	
Prefer Not to Answer	0 (0.00%)	0 (0.00%)	
I Don't Know	1 (4.17%)	0 (0.00%)	
BMI, Mean (SD)	28.80 (5.30)	31.50 (8.25)	0.20
FMS Score, Mean (SD)	5 (5.00)	22 (6.00)	<0.001*

Table 4. Materials used for RT-qPCR and luciferase assays.

Item Name	Company	Cat No.	Assay ID	Sequence
PAXgene Blood miRNA Kit	Qiagen	763134	N/A	N/A
TRIzol® Reagent	ThermoFisher	15596018	N/A	N/A
mirVana™ miRNA mimic, miR-133a-3p	ThermoFisher	4464066	MC10413	UUUGGUCCCCUUCAACCAGCUG
mirVana™ miRNA Inhibitor, Negative Control #1	ThermoFisher	4464076	N/A	Proprietary
mirVana™ miRNA Mimic, miR-1 Positive Control	ThermoFisher	4464062	N/A	UGGAAUGUAAAGAAGUAUGUAU
INTERFERin Transfection Reagent	PolyPlus	101000028	N/A	N/A
miR-133a-3p Probe	Qiagen	339306	YP00204788	UUUGGUCCCCUUCAACCAGCUG
cel-miR-39-3p Probe	Qiagen	339306	YP00203952	UAUACCGAGAGCCCAGCUGAUUUCGUCUUGGUAAUAAGCUCGUCAUUGAGAUUAUCACCGGGUGUAAAUCAGCUUGGCUCUGGUGUC
UniSp6 Probe	Qiagen	339306	YP00203954	Proprietary
miRCURY LNA RT	Qiagen	339340	N/A	N/A
miRNA Spike In	Qiagen	339390	N/A	N/A
miRCURY LNA SYBR Green PCR	Qiagen	339345	N/A	N/A
ATXN7 Probe	ThermoFisher	4331182	Mm01315281_m1	NM_139227.4
CRK Probe	ThermoFisher	4331182	Mm00467065_m1	NM_133656.5
HIF1A Probe	ThermoFisher	4331182	Mm00468869_m1	NM_010431.2
HSP90AA1 Probe	ThermoFisher	4331182	Mm00658568_gH	NM_010480
HSP90B1 Probe	ThermoFisher	4331182	Mm00441926_m1	NM_011631.1
IL-6 Probe	ThermoFisher	4331182	Mm00446190_m1	NM_031168.1
KIDINS220 Probe	ThermoFisher	4331182	Mm01287158_m1	NM_001081378.1
MAP3K3 Probe	ThermoFisher	4331182	Mm00803725_m1	NM_011947.3
NUMB Probe	ThermoFisher	4331182	Mm00477927_m1	NM_001272056.1
PTPN11 Probe	ThermoFisher	4331182	Mm00448434_m1	NM_001109992.1
TGFBR2 Probe	ThermoFisher	4331182	Mm00436976_m1	NM_009371.3
ACTB Probe	ThermoFisher	4331182	Mm02619580_g1	GenBank: AK075973.1
18S Probe	ThermoFisher	4331182	Mm03928990_g1	NR_003278.3
High-capacity cDNA Reverse Transcription Kit	ThermoFisher	4368813	N/A	N/A
TaqMan Fast Advanced Master Mix	ThermoFisher	4444557	N/A	N/A
CRK luciferase vector	GeneCopeia	MmiT091607-MT05	N/A	NM_133656.5

HSP90AA1 luciferase vector	GeneCopeia	MmiT029411-MT05	N/A	NM_010480.5
HSP90B1 luciferase vector	GeneCopeia	MmiT030394-MT05	N/A	NM_011631.1
IL1B luciferase vector	GeneCopeia	MmiT092978-MT05	N/A	NM_008361.4
MAP3K3 luciferase vector	GeneCopeia	MmiT149016-MT05	N/A	NM_011947.4
NUMB luciferase vector	GeneCopeia	MmiT093937-MT05	N/A	NM_001136075.2
Secrete-Pair Dual Luminescence Assay Kit	GeneCopeia	LF033	N/A	N/A

Table 5. Materials used for IF, IHC, ISH, and western blot.

Item Name	Company	Cat No.	Host Species	Class	Dilution
eGFP Antibody	ThermoFisher	OSE00003G	Rabbit/IgG	Polyclonal	1:500
GFAP Antibody	Dako	Z033401-2	Rabbit/Ig fraction	Polyclonal	1:1,000
IBA1 Antibody	ThermoFisher	OSE00003G	Rabbit/IgG	Polyclonal	1:500
MYH1 Antibody	ThermoFisher	PA5-117077	Rabbit/IgG	Polyclonal	1:200
NeuN Antibody	Sigma-Aldrich	634304	Guinea Pig	Polyclonal	1:500
Perilipin Antibody	Abcam	Abcam3526	Rabbit/IgG	Polyclonal	1:2,000
Glycerol Mounting Media with DAPI	Abcam	ab188804	N/A	N/A	N/A
AlexaFluor 488	ThermoFisher	A21206	Donkey/IgG	Polyclonal	1:500
AlexaFluor 594	Jackson ImmunoResearch	715-585-150	Donkey/IgG	Polyclonal	1:500
TSA Plus Cyanine 3 (Cy3)	Akoya Biosciences	NEL744001KT	N/A	N/A	1:500
TSA Plus Cyanine 5 (Cy5)	Akoya Biosciences	NEL745001KT	N/A	N/A	1:1,000
(H+L) Secondary, HRP Conjugated	ThermoFisher	31430	Goat/IgG	Polyclonal	1:1,000
GAPDH	Cell Signaling Technology	2118S	Rabbit/IgG	Monoclonal	1:1,000
Amersham ECL HRP-Linked	Cytiva	NA9340V	Goat/IgG	Polyclonal	N/A
Normal Donkey Serum	Jackson ImmunoResearch	017-00-121	Donkey	N/A	5-10%
Normal Goat Serum	Jackson ImmunoResearch	005-000-121	Goat	N/A	5-10%
RNAscope Plus Kit	ACD	322785	N/A	N/A	N/A
RNAscope Probe Dilutant	ACD	300041	N/A	N/A	N/A
RNAscope® TSA Buffer Pack	ACD	322810	N/A	N/A	N/A
RNAscope® Wash Buffer Reagents	ACD	310091	N/A	N/A	N/A
miR-133a-3p Probe (S1)	ACD	1327411-S1	N/A	N/A	N/A
CRK Probe (C2)	ACD	1209081-C2	N/A	N/A	N/A
HSP90AA1 Probe (C3)	ACD	1327481-C3	N/A	N/A	N/A
HSP90B1 Probe (C2)	ACD	556051-C2	N/A	N/A	N/A
MAP3K3 Probe (C4)	ACD	1311361-C4	N/A	N/A	N/A
NUMB Probe (C3)	ACD	473401-C3	N/A	N/A	N/A
RNAscope® Wash Buffer Reagents	ACD	310091	N/A	N/A	N/A
MAP3K3 Antibody	Cell Signaling Technology	5727T	Rabbit	Monoclonal	1:1,000

CRK Antibody	Cell Signaling Technology	8690T	Rabbit	Monoclonal	1:1,000
HSP90AA1 Antibody	Genuinbiotech.com	#3422	Mouse	Monoclonal	1:5,000
HSP90B1 Antibody	Selleckchem.com	F1132	Rabbit	Monoclonal	1:100,000
NUMB Antibody	Genuinbiotech.com	#65751	Rabbit	Monoclonal	1:5,000
P44/42 MAPK (ERK1/2) Antibody	Cell Signaling Technology	9102S	Rabbit	Polyclonal	1:1,000
