# Supplementary material for: Catecholamine-mediated release of miR-133a-3p from adipocytes regulates the onset of chronic primary pain
Source: J Clin Invest. 2026 Jul 21;136(17):e197345. doi: 10.1172/JCI197345 (PMC13528929; doi:10.1172/JCI197345)
Supplement: Supplementary file 2 [file jci-136-197345-s021.pdf]

Figure 7A

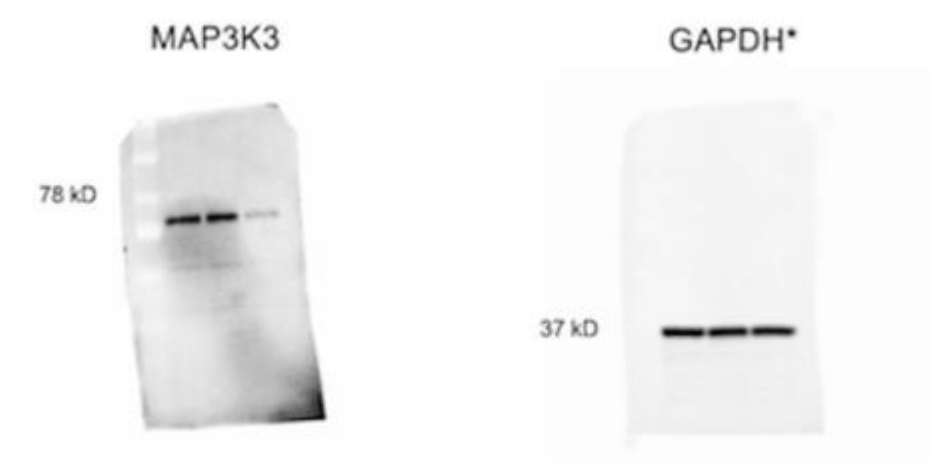

Figure 7B

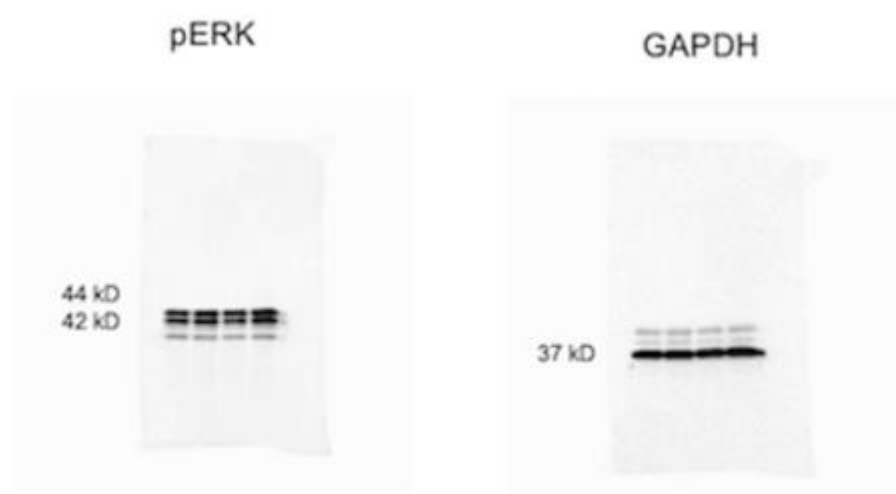

Supplemental Figure 11 Part 1

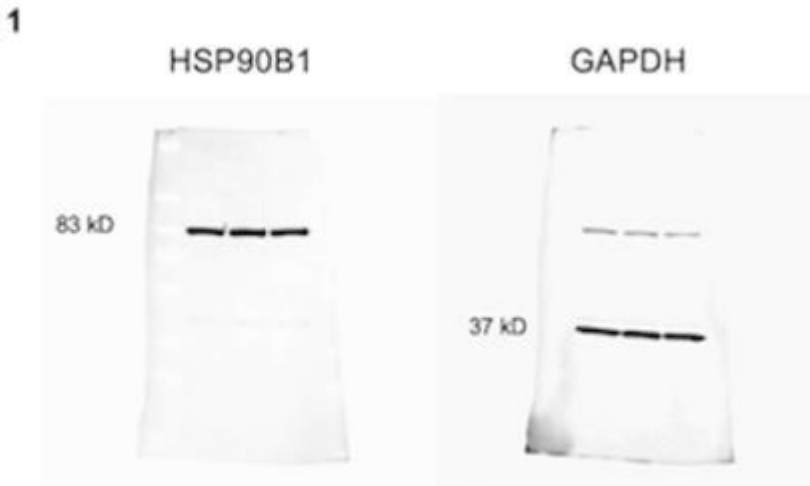

Supplemental Figure 11 Part 2

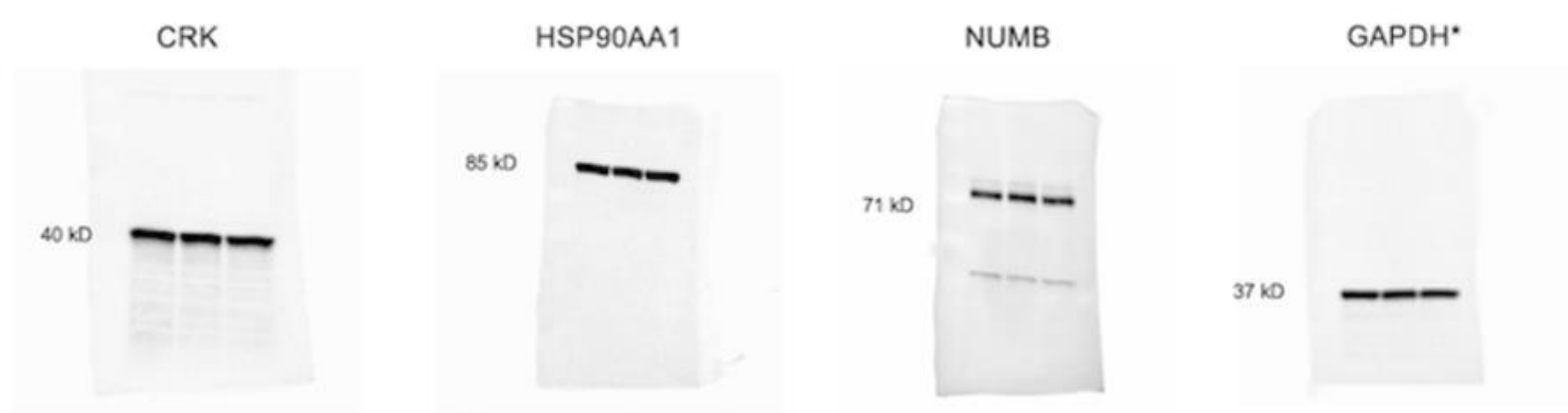

* = Same GAPDH from Figure 7A
